# Preterm Birth and the Development of Visual Attention During the First 2 Years of Life: A Systematic Review and Meta-analysis

**DOI:** 10.1001/jamanetworkopen.2021.3687

**Published:** 2021-03-30

**Authors:** Or Burstein, Zipi Zevin, Ronny Geva

**Affiliations:** 1 Department of Psychology, Bar Ilan University, Ramat Gan, Israel; 2 Gonda Multidisciplinary Brain Research Center, Bar Ilan University, Ramat Gan, Israel

## Abstract

**Importance:**

Preterm birth is associated with an increased risk for long-lasting attention deficits. Early-life markers of attention abnormalities have not been established to date but could provide insights into the pathogenesis of attention abnormalities and could help identify susceptible individuals.

**Objective:**

To examine whether preterm birth is associated with visual attention impairments in early life, and if so, in which attention functions and at which developmental period during the first 2 years of life.

**Data Sources:**

PubMed and PsycINFO were searched on November 17, 2019, to identify studies involving visual attention outcomes in infants born preterm vs full term.

**Study Selection:**

Peer-reviewed studies from the past 50 years met the eligibility criteria if they directly assessed visual attention outcomes until the age of 2 years in generally healthy infants born preterm or full term. The selection process was conducted by 2 independent reviewers.

**Data Extraction and Synthesis:**

The Meta-analysis of Observational Studies in Epidemiology (MOOSE) reporting guideline was followed. Random-effects models were used to determine standardized mean differences. The risk of bias was assessed both within and between studies.

**Main Outcomes and Measures:**

Five nascent indices of visual attention were analyzed, including very basic functions—namely, the abilities to follow and fixate on visual targets—and more complex functions, such as visual processing (ie, habituation), recognition memory (ie, novelty preference), and the ability to effortfully focus attention for learning.

**Results:**

A total of 53 studies were included, with 69 effect sizes and assessing a total of 3998 infants (2047 born preterm and 1951 born full term; of the 3376 for whom sex was reported, 1693 [50.1%] were girls). Preterm birth was associated with impairments in various attention indices, including visual-following in infancy (Cohen *d*, −0.77; 95% CI, −1.23 to −0.31), latency to fixate (Cohen *d*, −0.18; 95% CI, −0.33 to −0.02), novelty preference (Cohen *d*, −0.20; 95% CI, −0.32 to −0.08), and focused attention (Cohen *d*, −0.28; 95% CI, −0.45 to −0.11). In the neonatal period, preterm birth was associated with superior visual*-*following (Cohen *d*, 0.22; 95% CI, 0.03 to 0.40), possibly owing to the additional extrauterine exposure to sensory stimulation. However, this early association waned rapidly in infancy (Cohen *d*, –0.77; 95% CI, –1.23 to –0.31).

**Conclusions and Relevance:**

The findings suggest that preterm birth is associated with impingements to visual attention development in early life, as manifested in basic and then complex forms of attention. Advancements in neonatal care may underlie improvements found in the current era and accentuate several early protective factors.

## Key Points

**Question:**

Is preterm birth associated with visual attention impairments in early life, and if so, in which attention functions?

**Findings:**

This systematic review and meta-analysis of 53 studies including 2047 preterm-born and 1951 full-term–born neonates and infants found that preterm birth was significantly associated with impairments in visual attention functioning. Despite a short-term advantage in visual-following in preterm infants, deficits cascaded from basic orienting responses to focused attention during the first 2 years of life.

**Meaning:**

The findings suggest that preterm birth is associated with challenges in the development of visual attention beginning in the early stages of life.

## Teaser

This systematic review and meta-analysis investigates visual attention functioning during the first 2 years of life in infants born preterm versus full term.

## Introduction

Preterm birth, a live birth before gestational age (GA) of 37 weeks,^1^ is associated with substantial developmental challenges. Advancements in obstetrics and neonatal care have been associated with an increase in preterm births worldwide,^2^ which account for 10.6% of live births.^3^ Despite the positive aspects of better survival and care, preterm birth has implications for learning,^4^ cognitive performance,^4,5,6^ and attention^5^; deficits in these areas may underlie the association of preterm birth with poorer quality of life^7^ and substantial economic costs.^8,9^

Susceptibility to these long-term deficits may manifest during the first 2 years of life,^10,11^ which is a pivotal period for attention development involving the progression from basic abilities that are highly reactive and dependent on external cues^12^ to an initial exertion of volitionally channeled attention.^13^ It is important to understand how a congenital vulnerability (eg, preterm birth) affects this expected developmental course. Neurodevelopmental frameworks advocate a model that traces the long-term cognitive sequelae of preterm birth to the cascading implications of early dysfunctions in regulatory and attentional facets.^14,15,16^ This approach accentuates the idea that the emerging operations of higher cortical loci, which are later to materialize from both the phylogenetic and the ontogenetic perspectives, are influenced by earlier dysregulation of midbrain structures,^17,18^ thus compromising the distributed attentional networks further as the child matures.^19,20,21^ The stage-specific behavioral-attention expressions of this cascade are not yet fully mapped.

The third trimester of pregnancy is a period of rapid neural growth for the fetus.^22^ Younger GA at birth is associated with diminished myelinogenesis,^23^ synaptogenesis, and dendritic sprouting.^24^ Extrauterine exposure to sensory stimulation in premature neonates exposes the underdeveloped neural network to stressors, resulting in augmented sensitivity^25,26^; this marks the neonatal period as a supersensitive period for attention development. Thus, reviewing the formation of notable attention functions in the early life of infants born preterm may reveal factors needing consideration.

Basic attention faculties, typically available in the neonatal period, include the ability to fixate on salient cues in the periphery of the visual field^27^ and follow salient visual stimuli.^28^ These faculties further develop during the first year of life.^28,29^ After the transition from reliance on brainstem-basilar–mediated pathways to the increasing involvement of cortical structures in the second year of life, infants exert more endogenous direction over visuospatial attention.^13^ This is expressed by 2 notable abilities that predict intellectual performance in later childhood in both the typically developing and the preterm-born populations^10,11^: first, novelty preference (ie, the ability to preferentially attend to a novel stimulus),^12^ and then focused attention (ie, the ability to effortfully sustain attention to explore objects).^30^

To date, it remains unclear whether preterm birth triggers a negative cascading effect on attention development and how it affects each essential attention function in early life. Based on the cascade assertion, it is expected that deficits in more basic functions such as following and latency to fixate on visual stimuli will be evident in early developmental stages and might ebb in later stages; regarding endogenous attention functions such as novelty preference and focused attention, it is expected that earlier neurophysiological dysregulation will lead to long-lasting deficits.^13,15,20,21^ Vis-à-vis the implications of early extrauterine exposure to sensory stimulation, it is possible that during the first weeks of life, preterm infants will nevertheless benefit in precocial attention abilities resulting from the additional exercise of the visual system.^29^ However, it is conjectured that this early advantage at term age will rapidly wane, because the burden of premature stimulation and activation of attention networks will be associated with durable deficits in attention tasks from early infancy onward.^29^ To assess these hypotheses, a concise review of the literature is required.

During the past 50 years, a myriad of studies assessed the association between preterm birth and attention development in early life, but only a few reviews attempted to synthesize and generalize the findings.^29,31,32,33,34,35,36,37,38,39,40,41,42,43,44,45,46,47,48,49,50,51,52,53,54,55,56,57,58,59,60,61,62,63,64,65,66,67,68,69,70,71,72,73,74,75,76,77,78,79,80,81,82^ Two notable reviews^83,84^ from previous decades suggested that infants born preterm show less-optimal attention performance. However, because both reviews were nonsystematic and did not attempt a statistical synthesis, there was a necessity for a methodical review to revisit the important claims of the previous studies while assigning distinct emphasis to each of the focal-attention faculties.

We expected that the developmental course would be significantly associated with preterm birth in ways that cascade from basic to more endogenous forms of attention. The aim of this study was to examine which attention faculties might benefit from early extrauterine exposure to stimuli and which might be compromised as the preterm-born infant experiences increasing attentional demands. In addition, we aimed to assess whether the substantial advancements in neonatal care were also associated with ameliorations in attention development.

## Methods

### Systematic Review Protocol

In this systematic review and meta-analysis, we reviewed the literature to identify studies involving visual attention outcomes in infants born preterm vs full term. This study followed the Meta-analysis of Observational Studies in Epidemiology (MOOSE) reporting guideline.^85^

The eligibility criteria for included studies were (1) publication in a peer-reviewed scientific journal or book, (2) publication during the past 50 years, (3) inclusion of both a preterm (ie, GA<37 weeks at birth) and a full-term group, (4) inclusion of infants aged 2 years or younger, (5) inclusion of healthy participants (indicating that at least 75% of the preterm-born sample was reported to be without chronic neurological, genetic, or medical impairment at the time of assessment), and (6) reporting of at least 1 visual attention measure that had been directly attained from testing the participant (ie, questionnaires and indirect report indices were not eligible).

### Information Sources and Search Strategy

The literature review was conducted November 17, 2019, in the PubMed and PsycINFO databases using combinations of the following keywords: [*preterm* OR *premature* OR *pre-term* OR *prematurity*] AND *attention* AND [(*infant* OR *infants* OR *infancy*) OR (*neonate* OR *neonates* OR *neonatal*) OR (*toddler* OR *toddlers* OR *toddlerhood*)]. Further records were identified by manual review of studies’ references.

### Study Selection

Records were integrated into the Colandr platform,^86^ and duplicates were removed. Subsequently, titles and abstracts were screened. Full-text articles were assessed for eligibility according to the inclusion criteria. Reasons for exclusion were stored in the system. The entire selection process was conducted by 2 independent reviewers (O.B. and Z.Z.); conflicts (less than 10% of the cases) were resolved by dialogue.

### Data Collection

Data were collected and maintained using a sheet generated ad hoc. Apart from the outcome statistics, crucial covariates were extracted. The specific method and operational definition of the attention measures were documented (eTable 1 in the Supplement). The quality of the studies was screened using a modified version of the Newcastle-Ottawa Scale^87^ (eAppendix 1 in the Supplement).

### Statistical Analysis

Data were analyzed using the metafor package in R, version 3.6.1 (R Project for Statistical Computing).^88^ Because various studies used different scales to measure the same constructs, group differences were expressed as standardized mean differences with the Cohen *d* index.^89^ Random-effects models were used based on the DerSimonian-Laird method.^90^

Heterogeneity between studies was assessed using Cochrane *Q* and *I*^2^ statistics.^91^ For the *Q* statistic, 2-sided *P* < .10 was considered significant.^91^ For the *I*^2^ statistic, previously established guidelines were followed.^91^

Screening for potential outliers was conducted through inspection of the externalized studentized residuals.^92^ The risk of bias between studies was assessed by inspection of the degree of asymmetry of the funnel plot using Egger regression^93^ and by the trim-and-fill method.^94^ These assessments are presented in eFigure 1 in the Supplement.

The contribution of important demographic and medical factors was assessed using moderation analyses. Only significant moderators are reported (further details are provided in eAppendix 2 in the Supplement).

## Results

### Overall Characteristics of Studies

The selection process yielded 53 eligible studies,^29,31,32,33,34,35,36,37,38,39,40,41,42,43,44,45,46,47,48,49,50,51,52,53,54,55,56,57,58,59,60,61,62,63,64,65,66,67,68,69,70,71,72,73,74,75,76,77,78,79,80,81,82^ providing a total of 69 effect sizes distributed over 5 meta-analyses. The overall sample included 3998 neonates and infants (of the 3376 for whom sex was reported, 1693 [50.1%] were girls); 2047 were born preterm, and 1951 were full-term control individuals. The mean (SD) birthweight and GA at birth of the preterm populations were 1514 (458) g and 31.4 (2.5) weeks, respectively. The infants’ mean (SD) age at testing was 29.3 (22.8) weeks. The visual attention outcomes included (1) visual-following, (2) latency to fixate, (3) habituation, (4) novelty preference, and (5) focused attention. A flow diagram depicting the selection process is shown in eFigure 2 in the Supplement. A comprehensive description of the characteristics of the studies included in this meta-analysis are publicly available through an open data repository.^95^

### Visual-Following

**Figure 1.**
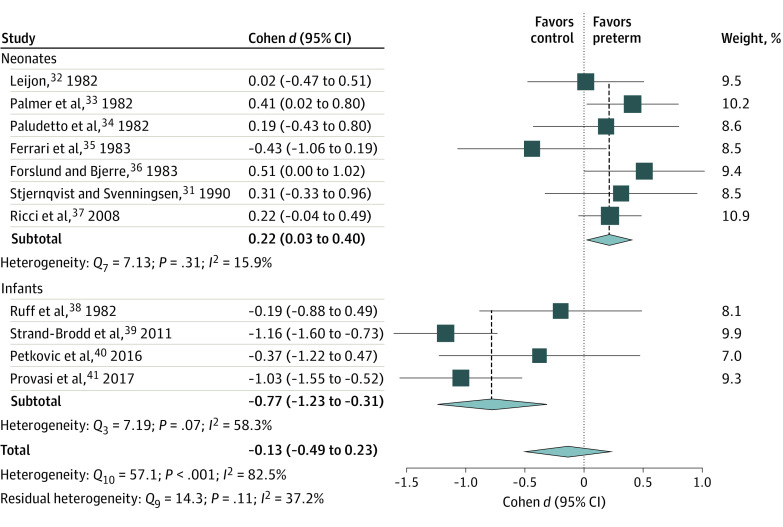
Forest Plot for the Differences in Visual-Following Between Infants and Neonates Born Preterm and Full-term Squares represent point estimates, with the marker size indicating weight; horizontal lines represent 95% CIs. Diamonds represent the pooled point estimate, with the points indicating the 95% CI.

Eleven eligible studies^31,32,33,34,35,36,37,38,39,40,41^ with 11 effect sizes on measures of visual following of an inanimate object were included (Figure 1). No differences were found between the full-term and preterm groups in visual-following (Cohen *d*, −0.13; 95% CI, −0.49 to 0.23). However, there was evidence of heterogeneity in effect sizes between studies (*Q*_10_ [subscript indicates degrees of freedom {*df*}], 57.08; *P* < .001), suggesting that a portion of the variance was explained by heterogeneity rather than chance (*I*^2^, 82.5%). Subgroup analysis indicated that the source of heterogeneity was associated with the infants’ age at the time of the test (*Q_moderator [M]_*, 23.14; *df*, 1; *P* < .001). A qualitative interaction was detected, suggesting that individuals born preterm were more likely to manifest superior performance during the neonatal period (Cohen *d*, 0.22; 95% CI, 0.03- 0.40) but inferior performance in early infancy (Cohen *d*, −0.77; 95% CI, −1.23 to −0.31).

No evidence of heterogeneity was found in the studies of neonates, but an indication of heterogeneity was found in the studies of infants (*Q*_3_, 7.19 [*P* = .06]; *I*^2^, 58.3%). A difference in effect sizes was found (*Q_M_*, 6.95; *df*, 1; *P* = .008) between studies that used real-time observers’ coding of gaze (Cohen *d*, –0.26; 95% CI, −0.80 to 0.27) and those that used computerized gaze tracking (Cohen *d*, –1.11; 95% CI, −1.44 to −0.77). These findings suggest a quantitative interaction, with a more robust difference between the groups when computerized equipment was used (plausibly owing to increased tracking sensitivity).

Taken together, the studies showed that neonates born preterm had a greater likelihood for advantage in following salient cues. However, soon after birth, this likelihood for advantage waned.

### Latency to Fixate

**Figure 2.**
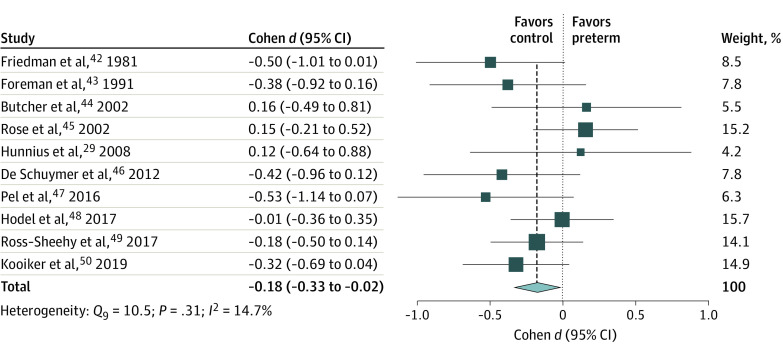
Forest Plot for the Differences in Latency to Fixate Between Infants Born Preterm and Full-term Squares represent point estimates, with the marker size indicating weight; horizontal lines represent 95% CIs. Diamonds represent the pooled point estimate, with the points indicating the 95% CI.

Ten eligible studies^29,42,43,44,45,46,47,48,49,50^ with 10 effect sizes on measures of latency to fixate on peripheral or salient stimuli were included (Figure 2). Preterm birth was associated with delayed latency to fixate (Cohen *d*, −0.18; 95% CI, −0.33 to −0.02; *z*, −2.15; *P* = .03). There was no evidence of heterogeneity. However, a moderation analysis indicated that birth era was associated with the differences (*Q_M_*, 6.38; *df*, 2; *P* = .041), suggesting an increased risk for deficits in preterm cohorts of infants born before 1990 and who are now at least 30 years of age (eAppendix 3 in the Supplement).

### Habituation and Novelty Preference

**Figure 3.**
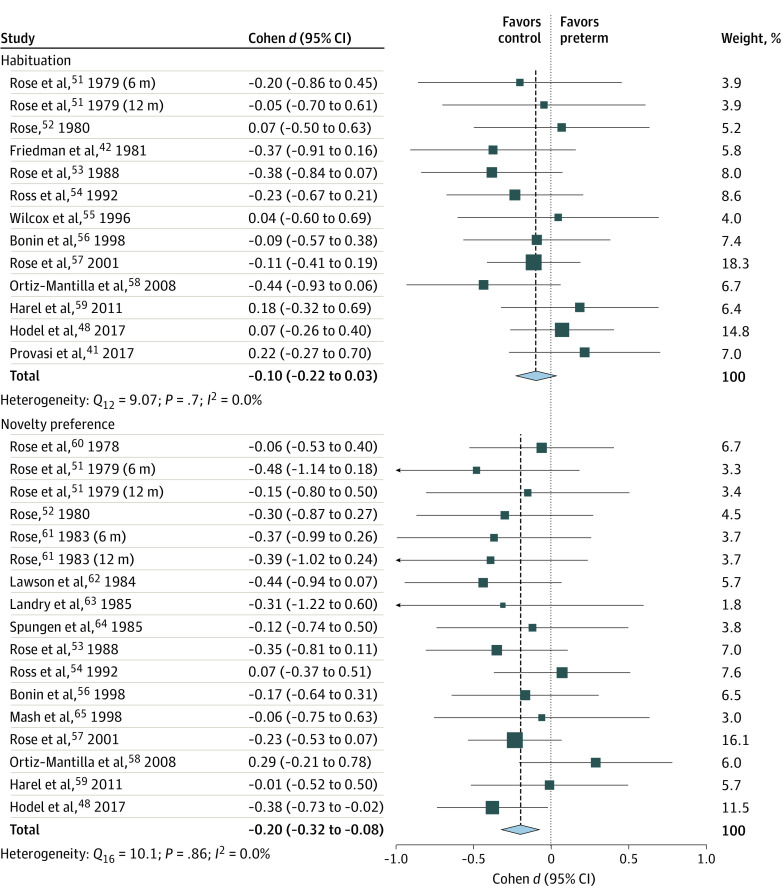
Forest Plot for the Differences in Habituation and Novelty Preference Between Infants Born Preterm and Full-term Squares represent point estimates, with the marker size indicating weight; horizontal lines represent 95% CIs. Diamonds represent the pooled point estimate, with the points indicating the 95% CI.

The constructs of habituation (the gradual decline in visual processing as a result of forming a mental representation of a stimulus) and novelty preference (the tendency to prefer exploring a novel stimulus rather than a familiar [ie, habituated] one) are theoretically intertwined because both reflect gaining familiarity with the stimulus through inspection. Both constructs were, therefore, considered in this review, and the association between them was analyzed (Figure 3).

For the meta-analysis of habituation, 12 eligible studies^41,42,48,51,52,53,54,55,56,57,58,59^ with 13 effect sizes were included. No differences were found between the groups in habituation (Cohen *d*, −0.10; 95% CI, −0.22 to 0.03). There was no evidence of heterogeneity. However, a moderation analysis indicated that birth era was associated with the differences (*Q_M_*, 4.77; *df*, 1; *P* = .03), suggesting an increased risk for deficits in preterm infants born before the year 2000 and who are now in their 20s (eAppendix 4 and eFigure 4 in the Supplement).

For the meta-analysis of novelty preference, 15 eligible studies^48,51,52,53,54,56,57,58,59,60,61,62,63,64,65^ with 17 effect sizes were included. Preterm birth was associated with diminished novelty preference (Cohen *d*, −0.20; 95% CI, −0.32 to −0.08; *z*, −3.21; *P* = .001). There was no evidence of heterogeneity.

To assess whether differences in habituation are associated with the extent of differences in novelty preference, we explored the 10 populations for whom both measures were reported. The analysis showed no association (β, −0.40; 95% CI, –1.20 to 0.39; *P* = .32).

Taken together, the findings showed that infants born prematurely were more likely to experience impairment in visual recognition memory as reflected in novelty preference, a difference that was also noticeable using the habituation paradigm in participants born before the year 2000.

### Focused Attention

**Figure 4.**
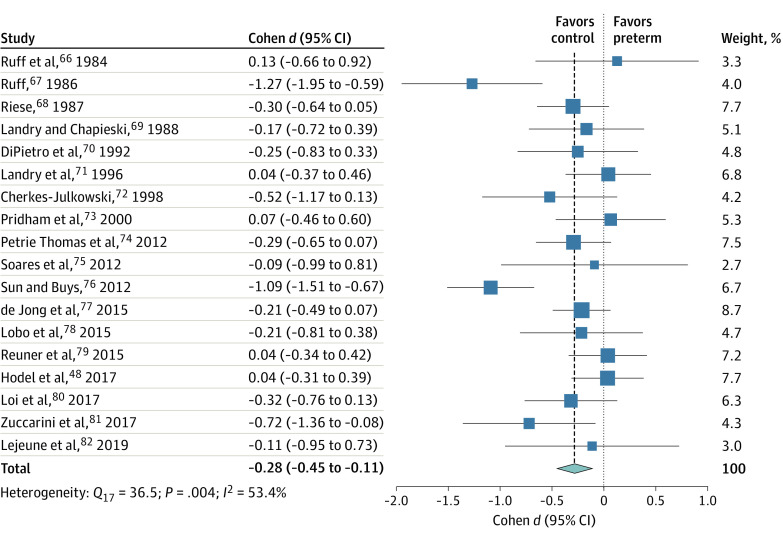
Forest Plot for the Differences in Focused Attention Between Infants Born Preterm and Full-term Squares represent point estimates, with the marker size indicating weight; horizontal lines represent 95% CIs. Diamonds represent the pooled point estimate, with the points indicating the 95% CI.

Eighteen eligible studies^48,66,67,68,69,70,71,72,73,74,75,76,77,78,79,80,81,82^ with 18 effect sizes on measures of intensified or deliberate orienting to an object were included (Figure 4). Preterm birth was associated with diminished focused attention (Cohen *d*, −0.28; 95% CI, −0.45 to −0.11; *z*, −3.25; *P* = .001). There was evidence of heterogeneity (*Q*_17_, 36.46; *P* = .004), indicating that a moderate portion of the variance was explained by heterogeneity rather than chance (*I*^2^, 53.4%). The heterogeneity was explained by 2 outlier findings; their removal attenuated the effect size but did not annul it (eAppendix 5 in the Supplement). A moderation analysis indicated an interaction between birth era and GA group (*Q_M_*, 11.65; *df*, 5; *P* = .04), suggesting that the increased risk for focused-attention deficits after extremely preterm birth was attenuated in cohorts born after 2000 but did not completely ebb (eAppendix 5 and eFigure 5 in the Supplement).

Taken together, the findings showed that in the multifaceted ability of focused attention, infants born preterm (especially extremely preterm) were more likely to experience difficulties compared with typically developing infants born at full term.

## Discussion

To our knowledge, this is the first systematic review and meta-analysis of preterm birth and the development of visual attention during infancy. Considering findings from 53 studies including 3998 infants, this review revealed that individuals born preterm had greater likelihood of attention difficulties as early as during the first 2 years of life. Increased likelihood for impairments cascaded from more reflexive functions—namely, visual-following (in early infancy but not during the neonatal period) and latency to fixate—to more endogenous forms of attention, such as visual recognition memory (as expressed by novelty preference but not by habituation), and was most apparent in focused attention. The current findings support findings from previous studies suggesting that the antecedents for the increased risk for diagnosis of attention deficit hyperactivity disorder and learning disabilities in individuals who were born at term^96^ and at preterm^4,5^ might already be discernible in infancy.

These differences were compatible with the hypothesized precocial exposure effect.^29,97^ Neonates born preterm were more likely to show an advantage in visual-following. It has been suggested that exposure to sensory stimulation after preterm birth might prime basic visual attention abilities.^29^ This claim is corroborated by the findings of this analysis. Preterm birth was associated with superior following even in a study of a population of extremely premature neonates with birthweight less than 901 g.^31^ An additional analysis (eFigure 3 in the Supplement) suggests that even though preterm birth was associated with superior visual-following to neutral stimuli, the visual system of preterm neonates was less primed to track human figures. An advantage in visual-following among neonates born preterm may not signal typical attention development; the current results suggest that this early advantage is likely to change in early infancy.

In measures of visual-following in infancy and latency to fixate, the meta-analyses showed that preterm infants had increased risk for deficits. Rudimentary ability to fixate on or follow salient visual targets is exogenous; retinal stimulation elicits activation of visual perception via the geniculate nucleus and its axonal oscillation of the primary visual cortex and oculomotor reaction via the superior colliculus.^98^ The findings from the current review and those of previous reports on children^99,100^ and adolescents^101^ concerning exogenous orienting suggest a neonatal advantage at term age, followed by a decline in infancy and then, putatively, recovery in exogenous orienting in childhood.

The recovery in exogenous orienting to stimuli is not corroborated by typical volitional orienting of attention. Preterm birth was associated with diminished performance in the reviewed early indices of endogenous attention (ie, novelty preference and focused attention). This finding complements the notion that preterm birth is associated with impeded development of endogenous attention throughout adolescence and adulthood.^102,103,104^ Focused attention—the hallmark of endogenous attention explored in the current meta-analysis—is contingent on executive substrates^105^ that undergo a maturational neural growth spurt at the approximate age of 10 months^106^ to facilitate the coordination of attention orienting and parasympathetic activation.^107^ Similarly, novelty preference relies on the dorsolateral prefrontal and anterior cingulate cortices that sustain attention and modulate arousal but also involves the hippocampus and parahippocampal cortex for encoding and decoding.^108^ Increased risk for alterations in the development of these regions in preterm-born populations^109,110^ further explains the difficulties in novelty preference. The risk for deficiencies in endogenous attention in infants born preterm that was observed in the current study thus implies the involvement of a widely distributed neural network that mostly involves the salience network.^111^ Unlike more reflexive attention abilities, deficits in endogenous or focused attention appear as a tenacious sequela of preterm birth^104^ that possibly initially manifest in the abilities to effortfully explore, process, and map the environment during infancy.

**Figure 5.**
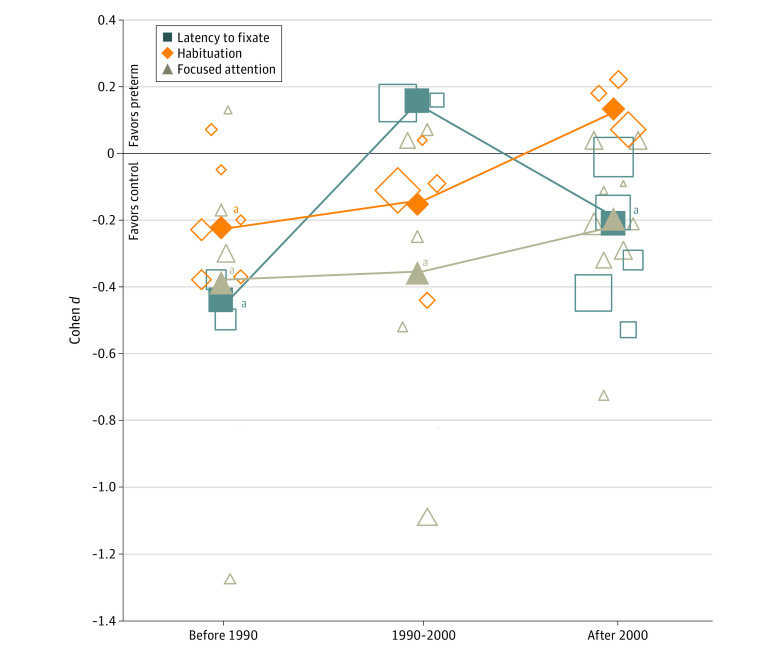
Differences in Visual Attention Development Between Infants Born Preterm and Full-term According to Birth Eras Outlined shapes represent effect sizes of distinct studies in the meta-analyses. Studies are separated according to the cohorts’ birth era. Filled shapes represent the standardized total mean difference between the groups in each birth era. The size of each faded shape is proportional to the study’s weight in the distinct meta-analysis. ^a^Significant difference (2-sided *P* < .05) between the full-term and preterm groups in a specific era.

The most promising finding from the present review in this regard involved signs that improvements in care and age-sensitive exposure were associated with attention outcomes. The current findings revealed a partial amelioration in focused-attention difficulties as a function of birth era and the accompanied revisions in care protocols (Figure 5). Two major revolutions have taken place in obstetrics and neonatal care in the past semi-century. The first was a medical revolution that occurred in approximately 1990^112^ and included the implementation of antenatal corticosteroid and postnatal surfactant treatments to diminish the risks for respiratory distress, brain damage, and mortality. The second was related to tailored sensorimotor stimulation, nurturance, and physio-emotional and social care during the neonatal stay and is manifested in excitation reduction, physical proximity to caregivers, and breastfeeding facilitation techniques.^113,114^ This analysis suggests that attention development during infancy may be sensitive to better-regulated oxygenation and well-titrated stimulation and that advancements in neonatal care may underlie the associated improvements in attention development by preventing overstimulation of posterior orienting and thereafter providing a stronger basis for the development of anterior executive attention networks.^115^ However, despite the positive trajectory, increased risk for early-life impairments in focused attention after extremely preterm birth still exists in the current era, suggesting that advancements in neonatal care may have enabled better prospects but that there is still more work to be done.

The findings support the notion that early development of attention skills may be sensitive to stimulation and care in ways that might merit future research: (1) extrauterine exposure to sensory stimulation may account for the short-term association with the precocial establishment of visual-following at term age, and (2) refinement of neonatal and pediatric care in the past 5 decades has improved remarkably in providing individually tailored medical, pharmacological, sensorimotor, emotional, thermal-physical, and social care. The findings of this review suggest that increased risk for deficits in volitional control over visual attention is associated with prematurity (and more pronouncedly with extreme prematurity). Advancements in care, mostly in the current era, seem to underlie a partial remission. Future developments in care during early infancy may support the ability of infants born preterm to focus attention to learn and fulfill their potential.

### Limitations

This study has limitations. First, a demographic bias resulting from overrepresentation of studies from Western Europe and North America (49 of the 53 included studies [92.3%]) curtailed the ecological validity of the findings vis-à-vis other nonrepresented populations. Second, some studies were missing information regarding central covariates (eg, 17.3% of the included studies were missing data on participants’ sex, 38.5% were missing information on participants’ age at the time of testing, and 79.0% were missing data on participants’ socioeconomic status). Reporting an adequate set of important background, demographic, and medical characteristics will likely improve the validity of future systematic reviews and deepen the understanding of the mechanisms involved.^116^

A third limitation was a lack of consistent reporting guidelines and the inaccessibility of data. When conducting an extensive literature review, attempts to reach authors in cases of insufficient data for calculation of effect sizes are not always successful. Most of the lost evidence in this review was from studies conducted in the 1970s, when there was less consensus on reporting guidelines.^117^ Adherence to acknowledged reporting guidelines may constrain this limitation in the future.^118,119^

## Conclusions

This systematic review and meta-analysis found that infants born preterm had increased risk for deficits in visual attention, cascading from basic reflexive functions (namely visual-following and latency to fixate) to difficulties in early operations of endogenous attention, such as novelty preference and focused attention, which are vital for learning about the world. The deficits were more pronounced in infants born extremely preterm. Advancements in neonatal care may underlie improvements found in the current era and accentuate several early protective factors.

## Notes

Supplement. **eFigure 1.** Funnel Plots of Effect Sizes and Standard Errors Used for the Trim and Fill Analyses for Assessing Possible Publication Biases **eFigure 2.** A Flow Diagram of Studies’ Selection Process **eFigure 3.** Forest and Funnel Plots for the Meta-Analysis on the Differences in Visual Following (Animate Stimuli) Between Preterm and Full-Term Born Neonates **eTable 1.** Characteristics of the Studies Included in the Meta-Analyses **eTable 2.** Preterm Birth and the Development of Visual Attention During the First Two Years of Life: A Systematic Review and Meta-analysis **eAppendix 1.** Modified Newcastle-Ottawa Scale for Assessment of Studies Quality and Risk of Bias **eAppendix 2.** Description of the Moderation Analyses **eAppendix 3.** Moderation Analysis in the Latency to Fixate Meta-analysis **eAppendix 4.** Moderation Analysis in the Habituation Meta-analysis **eFigure 4.** Graphical Depiction of the Moderation of the Differences Between the Preterm and Full-term Groups in Habituation by Birth Era **eAppendix 5.** Additional Analyses in the Focused Attention Meta-analysis **eFigure 5.** Graphical Depiction of the Moderation of the Differences Between the Preterm and Full-term Groups in Focused Attention by Birth Era and GA Group **eReferences**
