# Supplementary material for: Preterm Birth and the Development of Visual Attention During the First 2 Years of Life: A Systematic Review and Meta-analysis
Source: JAMA Netw Open. 2021 Mar 30;4(3):e213687. doi: 10.1001/jamanetworkopen.2021.3687 (PMC8010593; doi:10.1001/jamanetworkopen.2021.3687)
Supplement: Supplement. — eFigure 1. Funnel Plots of Effect Sizes and Standard Errors Used for the Trim and Fill Analyses for Assessing Possible Publication Biases eFigure 2. A Flow Diagram of Studies’ Selection Process eFigure 3. Forest and Funnel Plots for the Meta-Analysis on the Differences in Visual Following (Animate Stimuli) Between Preterm and Full-Term Born Neonates eTable 1. Characteristics of the Studies Included in the Meta-Analyses eTable 2. Preterm Birth and the Development of Visual Attention During the First Two Years of Life: A Systematic Review and Meta-analysis eAppendix 1. Modified Newcastle-Ottawa Scale for Assessment of Studies Quality and Risk of Bias eAppendix 2. Description of the Moderation Analyses eAppendix 3. Moderation Analysis in the Latency to Fixate Meta-analysis eAppendix 4. Moderation Analysis in the Habituation Meta-analysis eFigure 4. Graphical Depiction of the Moderation of the Differences Between the Preterm and Full-term Groups in Habituation by Birth Era eAppendix 5. Additional Analyses in the Focused Attention Meta-analysis eFigure 5. Graphical Depiction of the Moderation of the Differences Between the Preterm and Full-term Groups in Focused Attention by Birth Era and GA Group eReferences [file jamanetwopen-e213687-s001.pdf]

Supplementary Online Content

Burstein O, Zevin Z, Geva R. Preterm birth and the development of visual attention during the first 2 years of life: a systematic review and meta-analysis. *JAMA Netw Open*. 2021;4(3):e213687. doi:10.1001/jamanetworkopen.2021.3687.

eFigure 1. Funnel Plots of Effect Sizes and Standard Errors Used for the Trim and Fill Analyses for Assessing Possible Publication Biases
eFigure 2. A Flow Diagram of Studies' Selection Process
eFigure 3. Forest and Funnel Plots for the Meta-Analysis on the Differences in Visual Following (Animate Stimuli) Between Preterm and Full-Term Born Neonates
eTable 1. Characteristics of the Studies Included in the Meta-Analyses
eTable 2. Preterm Birth and the Development of Visual Attention During the First Two Years of Life: A Systematic Review and Meta-analysis
eAppendix 1. Modified Newcastle-Ottawa Scale for Assessment of Studies Quality and Risk of Bias
eAppendix 2. Description of the Moderation Analyses
eAppendix 3. Moderation Analysis in the Latency to Fixate Meta-analysis
eAppendix 4. Moderation Analysis in the Habituation Meta-analysis
eFigure 4. Graphical Depiction of the Moderation of the Differences Between the Preterm and Full-term Groups in Habituation by Birth Era
eAppendix 5. Additional Analyses in the Focused Attention Meta-analysis
eFigure 5. Graphical Depiction of the Moderation of the Differences Between the Preterm and Full-term Groups in Focused Attention by Birth Era and GA Group
eReferences

© 2021 Burstein et al. *JAMA Network Open*.

eFigure 1. Funnel plots of effect sizes and standard errors used for the trim and fill analyses for assessing possible publication biases

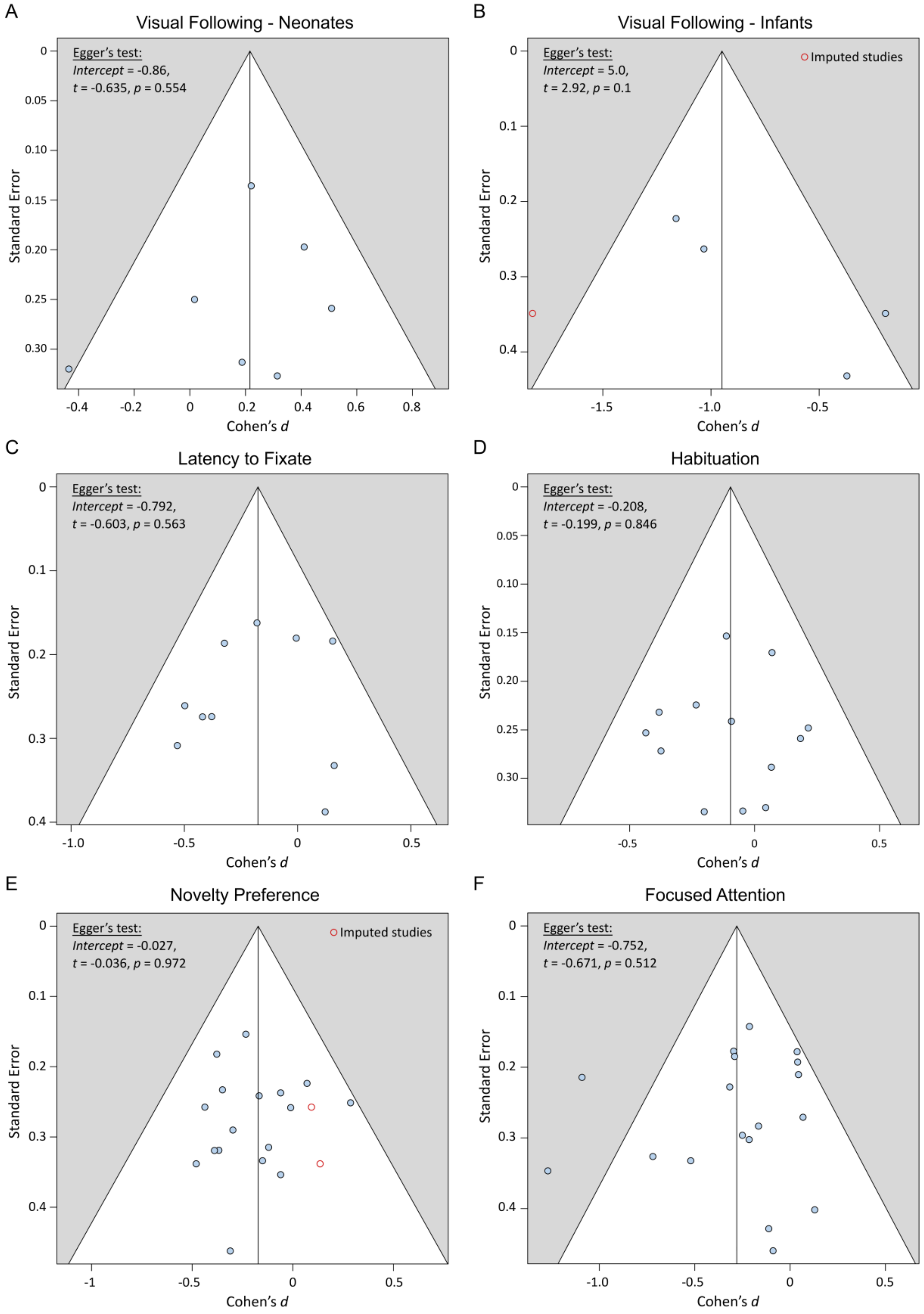

eFigure 1. (A) No indications of a publication bias were observed in the *Visual Following (Neonates)* meta-analysis. (B) Assessment of the funnel plot using the trim-and-fill method indicated a possible minor publication bias in the *Visual Following*

© 2021 Burstein et al. *JAMA Network Open*.

(Infants) meta-analysis, which led to the imputation of one additional study. The effect of this adjustment was negligible and slightly increased the total effect size, with an adjusted pooled estimate of -0.94 (95% CI, -1.43 to -0.45, $z = -3.77$, $p < 0.001$), suggesting that a publication bias could not better explain the result. (C) No indications of a publication bias were observed in the *Latency to Fixate* meta-analysis. (D) No indications of a publication bias were observed in the *Habituation* meta-analysis. (E) Assessment of the funnel plot using the trim-and-fill method indicated a possible minor publication bias in the *Novelty Preference* meta-analysis, which led to the imputation of two additional studies. The effect of this adjustment was negligible and slightly attenuated the total effect size, with an adjusted pooled estimate of -0.17 (95% CI, -0.29 to -0.06, $z = -2.92$, $p = 0.003$), suggesting that a publication bias could not better explain the result. (F) No indications of a publication bias were observed in the *Focused Attention* meta-analysis.

© 2021 Burstein et al. *JAMA Network Open*.

eFigure 2. A flow diagram of studies' selection process

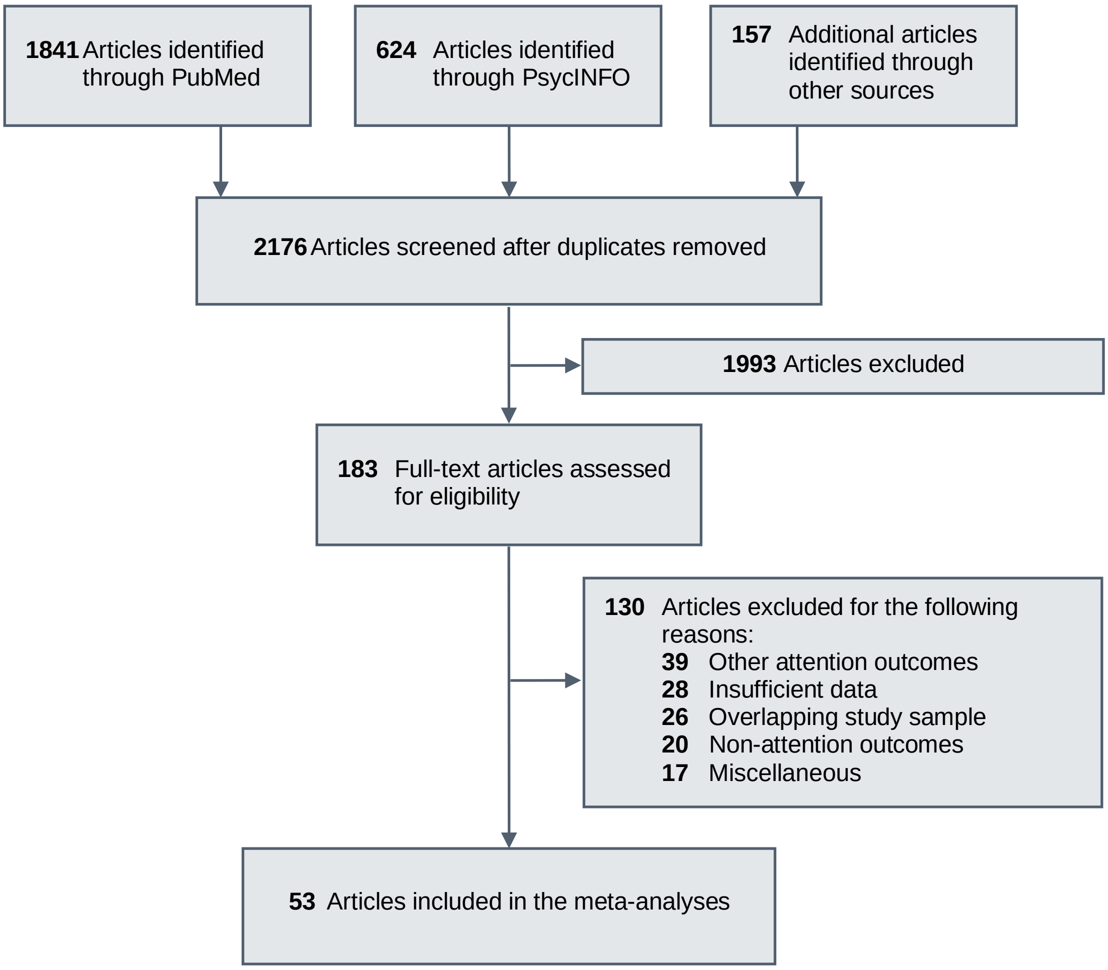

© 2021 Burstein et al. *JAMA Network Open*.

eFigure 3. Forest and funnel plots for the meta-analysis on the differences in visual following (animate stimuli) between preterm and full-term born neonates

eFigure 3A. A Forest Plot for the Differences in Visual Following (Animate Stimuli) Between Preterm and Full-term Born Neonates

Study	Cohen's *d* (95% CI)	Weight, %
Leijon,[22] 1982	-0.06 (-0.56 to 0.44)	30.0
Paludetto et al,[29] 1982	-0.37 (-0.98 to 0.25)	24.3
Ferrari et al,[9] 1983	-1.05 (-1.71 to -0.39)	22.5
Stjernqvist & Svenningsen,[53] 1990	-0.45 (-1.10 to 0.19)	23.2
**Total**	**-0.45 (-0.86 to -0.04)**	**100**

Q_3 = 5.56, *p* = 0.14, I^2 = 46%

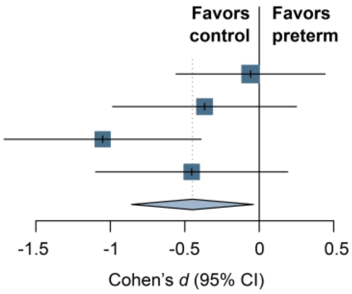

B

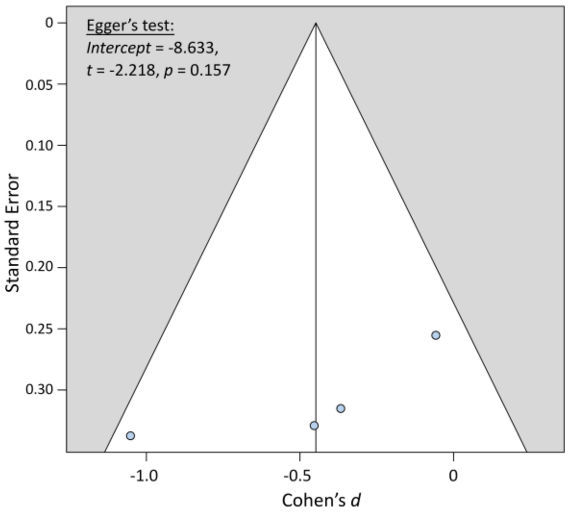

eFigure 3. (A) An additional analysis was conducted to address the four studies in neonates that also presented a measure of following an animate stimulus (*i.e.*, the examiner's face). The analysis indicated that in regard to socially-charged objects full-term neonates are more likely to present superior visual following, with a pooled effect size of -0.45 (95% CI, -0.86 to -0.04; z = -2.15, p = 0.03; k = 4, N = 218; Q_3 = 5.56, p = 0.14; I^2 = 46%). This implies that even though preterm-born neonates are more likely to show better reactiveness to neutral stimuli at term, their visual system is less primed to track human figures. However, this finding should be interpreted with caution due to the small number of studies and moderate evidence of heterogeneity. (B) A funnel plot of effect sizes and standard errors; no indication of a publication bias was found.

© 2021 Burstein et al. *JAMA Network Open*.

eTable 1. Characteristics of the studies included in the meta-analyses

Study	Included attention measure/s	Age in months	GA_{PT} (weeks) *M* (*SD*; Range)	BW_{PT} (g) *M* (*SD*)	Cohort	Setting	Exclusion criteria
Bonin et al,[1] 1998	**Novelty preference** PD<br>Ratio of looking at the novel stimulus to the looking time at the familiar and novel stimuli; stimuli: abstract patterns and naturalistic faces.<br>**Habituation** PD<br>Total looking time to reach criterion (infant-controlled); criterion: two consecutive fixations lasting 50% or less than the mean duration of the two longest fixations among the first three looks.	3.95L<br>(T1: 2.0;<br>T2: 3.92;<br>T3: 5.94)	32.1<br>(2.4; 27–36)	1712<br>(392)	Montreal, Canada	Lab	CNS disorder; congenital malformation syndromes; BPD; IVH > 1; 5' Apgar < 5; assisted ventilation > 28 d.
Butcher et al,[2] 2002	**Latency to fixate** PD<br>Latency to fixate (RT) and correct frequency on refixations on a peripheral stimulus; fixation stimuli: either shifting abstract shapes or a phase-reversing schematic face appearing on either the left or the right side of a monitor; peripheral target was a flashing shape; off-line scoring via video recordings; only noncompetition trials considered.	4L<br>(once a month from 6 to 26 weeks)	29.5<br>(1.0; < 32)	1183<br>(234)	Groningen, Netherlands 1995–1999	Lab	GA at birth > 32 weeks; serious neonatal medical complication according to the cutoff point of the NBRS; ROP; IVH > 1.
Cherkes-Julkowski,[3] 1998	**Focused attention**<br>Total amount of engaged time with the object (based on Krakow & Kopp,[4] 1983; "Sustained attention: engaged and occupied with toys"); seven min of free-play with age appropriate toys (last 6 min were coded).	14L<br>(T1: 13;<br>T2: 15)	36.3 (0.7)	1878<br>(848)	USA	Home	GA at birth > 37 weeks; BW > 2300 g; congenital disorder; ROP; NICU hospitalization after GA of 42 weeks; neurological impairment group was excluded.
de Jong et al,[5] 2015	**Focused attention**<br>"On-Task Persistence" (*i.e.*, consistent focus on activity; 5-point scale) during 5 min of free play with toys and 10 min of structured tasks (reading a book and making a puzzle with the mother).	17.3	34.7<br>(1.3; 32–36)	2585<br>(517)	Utrecht, Netherlands 2010–2011	Lab	Dysmaturity; multiple births; admission to a tertiary NICU; severe congenital malformations; maternal antenatal alcohol or drug use; chronic maternal antenatal use of psychiatric drugs.
De Schuymer et al,[6] 2012	**Latency to fixate**<br>Latency to first fixation (RT) on a peripheral stimulus (rectangles on a monitor); computerized eye-tracking; only noncompetition trials considered.	5.02L<br>(T1: 4.02;<br>T2: 6.01)	30.9<br>(1.4; 28–34)	1551<br>(406)	Ghent, Belgium	Home	IVH > 2; PVL; ROP > 2; abnormal Algo test.
DiPietro et al,[7] 1992	**Focused attention**<br>"Examining" (based on Ruff et al,[8] 1984); free play during 3 trials (2 min each).	7.67	30.2<br>(2.1; 26–33)	1409<br>(353)	USA	Lab	Congenital anomalies; maternal intravenous drug use; assisted ventilation > 10 d.
Ferrari et al,[9] 1983	**Visual following**<br>BNBAS: orienting to an inanimate visual stimulus (9-point scale); FT group assessed at the fifth PND; PT group assessed at a mean GA of 39.5 weeks.	Neonates	31.0<br>(2.4; 28–33)	1520<br>(250)	Modena, Italy 1978–1980	Hospital	1' Apgar < 5; 5' Apgar < 5; medial problems; abnormal somatic development; fetal distress; infection or sepsis; metabolic problem; RDS; CNS disorder; abnormal EEG functioning.
Foreman et al,[10] 1991	**Latency to fixate** PD<br>Latency (RT) to turn toward a peripheral stimulus (random check patterns); eight trials; coding by a single experienced observer (no videotape).	5.10<br>(range: neonates–12 m)	(25–34)	1590<br>(462)	Leicester, UK	Hospital	IVH; mild hydrocephalus; respiratory difficulties.
Forslund & Bjerre,[11] 1983	**Visual following**<br>Following the movement of a red ball (4-point scale); FT group assessed at a mean age of 3.6 days; PT group assessed at a mean GA of 40.2 weeks.	Neonates	~32.0 (< 35)		Malmö, Sweden 1976–1977	Hospital	

© 2021 Burstein et al. *JAMA Network Open*.

**eTable 1.** Characteristics of the studies included in the meta-analyses							
**Study**	**Included attention measure/s**	**Age in months**	**GA_{PT} (weeks) *M* (*SD*; Range)**	**BW_{PT} (g) *M* (*SD*)**	**Cohort**	**Setting**	**Exclusion criteria**
Friedman et al,[12] 1981	**Habituation**<br>Only visual condition; stimuli: green/red lighted box; two measures were considered: "Time to Response Decrement" and "Amount of Response decrement" indicating the time to reach decrement criterion (infant-controlled), and the difference in visual attention between the first trials and the last trial; both FT and PT groups were assessed at a mean GA of 40 weeks.<br>**Latency to fixate**<br>"Quickness of response" in the visual condition; latency (RT) to turn to the stimulus presented above as reflected on the pupils.	Neonates	33.7 (2.2)	1819 (388)	Washington, USA 1977–1979	Hospital	Abnormal BW for GA; poor medical history; inadequate rearing conditions.
Harel et al,[13] 2011	**Novelty preference**<br>Ratio of looking at the novel stimulus to the total looking time; one trial; stimuli: abstract patterns presented on a computer screen.<br>**Habituation**<br>Total looking time to reach criterion (fixed fixation time); criterion: accumulated 10 sec of looking at the stimulus.	3.29	32.2 (1.4; < 34)	1752 (364)	Ramat Gan, Israel	Home	High medical risk; chromosomal anomalies; IVH > 2.
Hodel et al,[14] 2017	**Novelty preference**<br>Difference in cumulative looking-times for the novel and familiar stimuli; stimuli: naturalistic faces presented on a monitor (same in habituation test); video coding of gaze direction (same in habituation and latency to fixate tests).<br>**Focused attention** [Cor]<br>Duration of visual attention toward a toy, including focused face and/or examining behaviors (based on Ruff et al,[15] 1990); one trial (2 min) of free play with a toy.<br>**Habituation**<br>Trials to reach criterion (infant-controlled); criterion: mean duration of looking for three consecutive fixations < 50% than the mean duration for the first three trials.<br>**Latency to fixate**<br>Latency (RT) to shift attention from a central to a peripheral stimulus; stimuli: colorful line drawings presented on either the left or right edges of a monitor.	9.13	35.0 (1.7; 30–36)	2467 (542)	MN, USA 2014	Lab	Neurological insult or disease; intrauterine growth restriction; congenital heart disease; serious medical illness.
Hunnius et al,[16] 2008	**Latency to fixate** [PD]<br>Latency to fixate (RT) and frequency of correct refixations on a peripheral stimulus; stimuli: either an abstract or a naturalistic face video appearing on either the left or the right side of a monitor; off-line scoring via a video recording; only noncompetition trials considered.	4[L] (once a month from 6 to 26 weeks)	29.6 (1.8; 27–32)	1267 (468)	Groningen, Netherlands 2000–2002	Lab	GA at birth > 32 weeks; prolonged ventilation; hemorrhagic and ischemic brain lesions; serious infections; ROP > 1.
Kooiker et al,[17] 2019	**Latency to fixate** [Med.1]<br>Reaction time to fixation (RT) and correct detection of salient targets; five visual stimuli (*i.e.*, cartoon, form, color, contrast and motion); gaze recorded via a computerized eye-tracking system.	12.1	27.6 (1.8; 24–32)	1038 (293)	Rotterdam, Netherlands 2010–2014	Lab	GA at birth > 33 weeks; visual acuity < 0.15; ROP > 3.

© 2021 Burstein et al. *JAMA Network Open*.

eTable 1. Characteristics of the studies included in the meta-analyses

Study	Included attention measure/s	Age in months	GA_{PT} (weeks) *M* (*SD*; Range)	BW_{PT} (g) *M* (*SD*)	Cohort	Setting	Exclusion criteria
Landry & Chapieski,[18] 1988	**Focused attention**<br>Mean length of time an infant spent looking at and/or physically examining a toy (based on Ruff et al,[8] 1984); one trial (2 min) of free play with a toy.	6	30.3 (1.6)	1205 (235)	Texas, USA	Lab	Low-risk group: moderate to severe degrees or clear precursors of cerebral palsy; sensory handicaps; non-IVH-related forms of hydrocephalus; IVH > 2; BPD.
Landry et al,[19] 1985	**Novelty preference**<br>Difference in the amount of time spent looking at the familiar stimulus (trial 7) vs. the novel stimulus (trial 8); stimuli: abstract patterns slides projected on a screen.	7	30.3 (2.5; 27–34)	1296 (260)	Texas, USA 1980–1981	Lab	No IVH group: sensory handicaps; diagnosis of cerebral palsy; non-IVH-related forms of hydrocephalus; IVH.
Landry et al,[20] 1996	**Focused attention**<br>Frequency of toy manipulation events during a 5 min toy-centered play with their mother.	6	31.0 (1.7; < 34)	1250 (248)	Texas, USA	Lab	Low-risk group: severe respiratory complications; IVH > 2.
Lawson et al,[21] 1984	**Novelty Preference**<br>Difference in time looking at the novel vs. the familiar stimulus on the visual-only test trial (simultaneous presentation); stimuli: objects moving on a track; gaze direction measured online using an event recorder.	2.93	31.7 (1.6)	1200 (201)	New York, USA	Lab	
Leijon,[22] 1982	**Visual following**<br>BNBAS: orienting to an inanimate visual stimulus (9-point scale); FT group assessed at the fifth PND; for the PT group only the assessment at term age (*i.e.*, GA of 39–41 weeks) was considered.	Neonates	30.3 (1.8; < 35)	1550 (323)	Linköping, Sweden	Hospital	Abnormal BW for GA; serious obstetrical problems; respiratory symptoms 48 h after birth; severe infections or hyperbilirubinemia; nutritional problems; inadequate postnatal growth; neurological symptoms; abnormal neurological function up to 18 m of age.
Lejeune et al,[23] 2019	**Focused attention**<br>"Sustained attention": reflecting the intensity of facial interest, duration of looking and duration of manipulation in a 3 min free play with decorated cubes (every 10 sec were coded on a 4-point scale, and then averaged for a composite score).	14.4	29 (2.2; < 32)	1207 (274)	Geneva, Switzerland 2013-2015	Hospital	Preterm control group: Major brain lesions detected on MRI (*e.g.*, IVH > 2)
Lobo et al,[24] 2015	**Focused attention**<br>"Looking while acting": infant's eyes directed toward the object while exhibiting another behavior (frequency); seven trials of free play with 7 separate objects (each trial up to 30 sec).	8.6[L] (nine times between birth and 2 years)	26.6 (0.4; 22–30)	881 (57)	Delaware, USA	Home	PT no BI group: PVL; IVH > 2
Loi et al,[25] 2017	**Focused attention**<br>"Sustained attention with objects": the degree to which the child engaged in focused and elaborative play during a 15 min play session with toys in the presence of their caregiver (7-point scale).	22.2	29.7 (1.9; 25–33)	1263 (308)	CA, USA 2008–2013	Lab	Bilingual environment; medical conditions that might preclude participation in the study.
Mash et al,[26] 1998	**Novelty preference**<br>Ratio of looking at the novel stimulus to the looking time at the familiar and novel stimuli (only Experiment 1 considered); four trials; stimuli: black press-on elements on white cards (above vs. bellow patterns); simultaneous presentation of familiar and novel stimuli; fixation times	3.66	31.7 (2)	1741 (460)	PA, USA	Lab	Mechanical ventilation > 7 d; BPD; CNS malformations; IVH > 2; progressive hydrocephalus; meningitis; cyanotic cardiac malformations; multiple congenital abnormalities; ROP; strabismus; nystagmus; optic atrophy.

© 2021 Burstein et al. *JAMA Network Open*.

**eTable 1.** Characteristics of the studies included in the meta-analyses							
**Study**	**Included attention measure/s**	**Age in months**	**GA_{PT} (weeks) *M* (*SD*; Range)**	**BW_{PT} (g) *M* (*SD*)**	**Cohort**	**Setting**	**Exclusion criteria**
	assessed online via corneal reflection.						
Ortiz-Mantilla et al,[27] 2008	**Novelty preference** Ratio of looking at the novel stimulus to the looking time at the familiar and novel stimuli (only visual task considered); stimuli: naturalistic faces; simultaneous presentation of familiar and novel stimuli; gaze direction coded online using a video feed. **Habituation** Trials to reach criterion (infant-controlled); criterion: mean duration of looking for two consecutive trials < 50% than the mean duration for the first two trials.	8.15[L] (T1: 6.65; T2: 9.65)	26.9 (2.2; 23–32)	976 (245)	New Jersey, USA 1996–1999	Lab	Primarily classified as low-risk infants.
Palmer et al,[28] 1982	**Visual following** Visual orientation to a red ball (Dubowitz method; 5-point scale); for the FT group the assessment at the fifth PND was considered; for the PT group, the assessment at term age was considered.	Neonates	31.5 (2.3; 27–35)	765–2490	London, UK 1979	Hospital	
Paludetto et al,[29] 1982	**Visual following** [Med.2] BNBAS: orienting to an inanimate visual stimulus (9-point scale); FT group assessed at the third PND; PT group assessed at a mean GA of 39 weeks.	Neonates	33 (27–36)	1723	Naples, Italy	Hospital	GA at birth > 37 weeks; abnormal BW for GA; Apgar 5' < 8; hyperbilirubinemia; abnormal glycaemia; neurological damage; serious attacks of apnea; sepsis; serious respiratory distress.
Pel et al,[30] 2016	**Latency to fixate** [Med.1] Reaction time to fixation (RT) and successful detection of salient targets (yes / no); five visual stimuli (*i.e.*, cartoon, form, color, expansion and motion); gaze recorded via a computerized eye-tracking system.	12.1	27.2 (1.1; < 29)		Rotterdam, Netherlands 2010–2013	Lab	Congenital malformations; whiter matter or gray matter damage (detected by MRI); visual acuity < 0.15; ROP > 3.
Petkovic et al,[31] 2016	**Visual following** Visual tracking of a black and white disc in horizontal, vertical and diagonal directions.	5	35.0 (1.3; 33–36)	2261 (382)	Zagreb, Croatia	Lab	IVH > 1; visual or auditory impairment; major neurological complications.
Petrie Thomas et al,[32] 2012	**Focused attention** Looking with a deliberate manipulation of the object, serious facial expression and a general decrease in extraneous body movements (based on Ruff & Lawson,[33] 1990; and Lawson & Ruff,[34] 2001); six min of free play with 4 toys (90 sec each); both duration and global (5-point scale) focused attention were considered.	8.06	29.2 (2.7; 23–32)	1322 (461)	Vancouver, Canada 2001–2004	Lab	Major congenital anomalies; IVH > 2; PVL; maternal antenatal drug use; abnormal BW.
Pridham et al,[35] 2000	**Focused attention** Looking and either holding or manipulating the toy (based on Ruff) during a 2 min toy-centered play with their mother designated for coding.	8	29.2 (2.7; < 33)	1220 (92)	WI, USA 1995–1998	Home	RDS group: IVH > 2; BPD
Provasi et al,[36] 2017	**Habituation** Reached criterion (yes/no); and trials and time to reach criterion (infant-controlled); criterion: mean duration of looking for three consecutive trials < 50% than the mean duration for the first three trials; stimulus: green bouncing	4.27	34.4 (0.9; 31–36)	1974 (372)	Paris, France	Lab	History of medical complications; prolonged ventilation; hemorrhagic and ischemic brain lesions; serious infections; visual impairments.

© 2021 Burstein et al. *JAMA Network Open*.

**eTable 1.** Characteristics of the studies included in the meta-analyses							
**Study**	**Included attention measure/s**	**Age in months**	**GA_{PT} (weeks)** ***M*** **(*SD*; Range)**	**BW_{PT} (g)** ***M*** **(*SD*)**	**Cohort**	**Setting**	**Exclusion criteria**
	ball on a computer screen; computerized eye tracking.<br>**Visual following** [PD]<br>Total looking time on ball course according to areas of interest; only synchronized trials considered.						
Reuner et al,[37] 2015	**Focused attention**<br>Time of intense looking and/or manipulating the toy, with high interest (based on Ruff et al,[15] 1990) during one minute of free play with three toys (20 sec each).	7.90	31.9<br>(3.1; 23–36)	1860<br>(698)	Heidelberg, Germany<br>2008–2009	Lab	Congenital anomalies; major sensory impairment; PVL; IVH > 2; other neurological complications; maternal antenatal drug use.
Ricci et al,[38] 2008	**Visual following** [PD]<br>Quality of eye tracking of an object; items 3-7 (*i.e.*, horizontal, vertical, arc and tracking a colored stimulus); FT group assessed at 48 hours after birth; PT group assessed at GA of 35 and 40 weeks (only second assessment was considered).	Neonates	28.2<br>(1.5; 25–31)	1155<br>(262)	Rome and Milan,<br>Italy<br>2004–2007	Hospital	GA at birth other than 25–31; abnormal cranial ultrasound; unstable clinical condition at GA of 35; oxygen-dependence at term; major congenital malformations; genetic chromosomal abnormality; metabolic disorders; congenital infection; signs of encephalopathy or seizures during neonatal course; jaundice requiring phototherapy; ROP > 2.
Riese,[39] 1987	**Focused attention**<br>"Attentiveness": indicating more focused or sustained attention (infant was alert to and maintained attention on the toy; 9-point scale); offline coding of a videotaped 2 min episode of free play with a mechanical toy.	24	34.0 (29–37)		KY,<br>USA	Lab	Twins study
Rose et al,[40] 1978	**Novelty preference**<br>Ratio of looking at the novel stimulus to the looking time at the familiar and novel stimuli (only intramodal/visual tasks considered); <u>stimuli:</u> objects; simultaneous presentation of familiar and novel stimuli (3 trials; 20 sec each) after a 30 sec familiarization trial (without habituation criterion).	12.4	32.6 (2.5)	1651<br>(303)	New York,<br>USA	Lab	Visual or neurological abnormalities.
Rose et al,[41] 1979	**Novelty preference**<br>Ratio of looking at the novel stimulus to the looking time at the familiar and novel stimuli (only visual task considered); <u>stimuli:</u> objects in different shapes; simultaneous presentation of familiar and novel stimuli for 20 sec.<br>**Habituation**<br>Time to reach criterion (fixed fixation time); <u>criterion:</u> accumulated 20 sec of looking at the stimulus.	<u>6 m:</u> 6.72<br><u>12 m:</u> 12.4	<u>6 m:</u> 33.4 (1.6)<br><u>12 m:</u> 33.2<br>(2.4)	<u>6 m:</u><br>1610<br>(271)<br><u>12 m:</u><br>1738<br>(250)	New York,<br>USA	Lab	Visual or neurological abnormalities.
Rose,[42] 1980	**Novelty preference**<br>Difference in time looking at the novel vs. the familiar stimulus; <u>stimuli:</u> abstract patterns, multidimensional variations of black lines on a white background and naturalistic faces (3 trials; 10 sec each); simultaneous presentation of familiar and novel stimuli.<br>**Habituation**<br>Time to reach criterion (fixed fixation time); <u>criterion:</u> accumulated 5, 10 or 20 sec of looking at the stimulus.	6.33	33.0 (2.4)	1633<br>(347)	New York,<br>USA<br>1974–1976	Lab	<u>Only non-intervention group:</u> visual or neurological abnormalities.
Rose,[43] 1983	**Novelty preference**	<u>6 m:</u>	<u>6 m:</u> 34.5 (1.9)	<u>6 m:</u>	New York,	Lab	Visual or neurological abnormalities.

© 2021 Burstein et al. *JAMA Network Open*.

eTable 1. Characteristics of the studies included in the meta-analyses							
**Study**	**Included attention measure/s**	**Age in months**	**GA_{PT} (weeks) *M* (*SD*; Range)**	**BW_{PT} (g) *M* (*SD*)**	**Cohort**	**Setting**	**Exclusion criteria**
	Ratio of looking at the novel stimulus to the looking time at the familiar and novel stimuli (simultaneous presentation); stimuli: four pairs of geometric forms (4 trials; 20 sec each); habituation: 10, 15, 20 or 30 sec accumulated looking time (infant-controlled).	6.49<br>12 m: 12.9	12 m: 34.6 (2.3)	1802 (450)<br>12 m: 1820 (381)	USA		
Rose et al,[44] 1988	**Novelty preference**<br>Ratio of looking at the novel stimulus to the looking time at the familiar and novel stimuli (simultaneous presentation); stimuli: abstract patterns, naturalistic faces and geometric forms (9 trials; either 10 or 20 sec each).<br>**Habituation**<br>Time to reach criterion (fixed fixation time); criterion: accumulated 5 or 20 sec of looking at the stimulus.	7	31.4 (1.7; 29–36)	1184 (211)	New York, USA 1979–1981	Lab	Congenital, neurological or physical abnormalities; BW > 1500 g.
Rose et al,[45] 2001	**Novelty preference**<br>Ratio of looking at the novel stimulus to the looking time at the familiar and novel stimuli (simultaneous presentation); stimuli: abstract patterns and naturalistic faces (9 trials; 10 sec each).<br>**Habituation**<br>Time to reach criterion (fixed fixation time); criterion: accumulated 3, 5, 10 or 20 sec of looking at the stimulus.	8.49[L] (T1: 5.24; T2: 7.80; T3: 12.9)	29.6 (2.9; 25–36)	1108 (283)	New York, USA 1994–1997[#]	Lab	Congenital, neurological or physical abnormalities; BW > 1750 g.
Rose et al,[46] 2002	**Latency to fixate**<br>Latency to fixate on a peripheral stimulus (RT; only baseline condition considered); stimuli: computer-generated images presented on either the left or right edges of a monitor; off-line scoring via a video recording.	8.49[L] (T1: 5.24; T2: 7.80; T3: 12.9)	29.6 (2.9; 25–36)	1108 (283)	New York, USA 1994–1997[#]	Lab	Congenital, neurological or physical abnormalities; BW > 1750 g.
Ross et al,[47] 1992	**Novelty preference**<br>Increase in % time looking at the novel stimulus (presented after a habituation stimulus); stimuli: schematic vs. scrambled face.<br>**Habituation**<br>Trials to reach criterion (infant-controlled); criterion: mean duration of looking for three consecutive trials < 50% than the mean duration for the first three trials with the schematic face.	10	30.35 (1.4; 28–32)	1463 (242)	New York, USA	Lab	Congenital malformations; moderate to severe neurosensory deficits; maternal antenatal drug use; abnormal weight for GA.
Ross-Sheehy et al,[48] 2017	**Latency to fixate** [PD]<br>Latency (RT) to shift attention from a central to a peripheral stimulus and frequency of correct responses (all cue conditions considered); stimuli: colorful images of everyday objects; off-line scoring via video recordings.	7.47 (range: 2–14)	31.8 (< 37)	1963	Iowa, USA	Lab	
Ruff et al,[49] 1982	**Visual following**<br>Time looking at the moving object; stimuli: three-dimensional crosses (either large or small) or a doll's head (either fast or slow) moving on a horizontal track (10 trials); gaze direction measured online using an event recorder.	1.49[L] (T1: 1.02; T2: 1.96)	31.5 (29–36)	< 1500	New York, USA	Lab	Both preterm groups considered: BW > 1500 g.
Ruff et al,[8] 1984	**Focused attention**	8.96	31.7 (26–34)	1217	New York,	Lab	Only low-risk group: BW > 1500 g.

© 2021 Burstein et al. *JAMA Network Open*.

**eTable 1.** Characteristics of the studies included in the meta-analyses							
**Study**	**Included attention measure/s**	**Age in months**	**GA_{PT} (weeks) *M* (*SD*; Range)**	**BW_{PT} (g) *M* (*SD*)**	**Cohort**	**Setting**	**Exclusion criteria**
	Exploratory behaviors (*i.e.*, alternating, rotating, fingering and transferring) during free play with toys (12 trials; each with a different object; 30 sec each).				USA		
Ruff,[50] 1986	**Focused attention**<br>Frequency of "examining" (*i.e.*, looking accompanied by fingering or turning the object with an intent facial expression; 6 trials; each with a different toy; 1 min each).	7.22	31.6	1198	New York, USA	Lab	BW > 1500 g.
Soares et al,[51] 2012	**Focused attention** [Med.1]<br>Frequencies of exploratory actions (based on Ruff et al,[8] 1984; fingering, transferring, rotating and alternating were considered); two trials (120 sec each) of free play with three soft rubber toys.	5.56[L]<br>(T1: 4.62;<br>T2: 5.57;<br>T3: 6.48)	35.6<br>(0.5; 32–36)	2960<br>(320)	São Paulo, Brazil	Lab	Prenatal complications; risk of cerebral palsy (*i.e.*, PVL, IVH, alterations in cerebral ultrasound); cardiopulmonary problems; hyperbilirubinemia; ROP; genetic syndromes; BW < 1750 g.
Spungen et al,[52] 1985	**Novelty preference**<br>The difference in the amount of time spent looking at the familiar (target 1, last trial) vs. the novel stimulus (target 2, first trial); stimuli: abstract patterns; FT group assessed between the first and the third PND; PT group assessed at a mean GA of 41.3 weeks.	Neonates	32.0<br>(3.1; 28–40)	1219<br>(354)	New York, USA	Hospital	Neurological abnormalities and sickness at the time of testing.
Stjernqvist & Svenningsen,[53] 1990	**Visual following**<br>BNBAS: orienting to an inanimate visual stimulus (9-point scale); FT group assessed at a mean GA of 40.6 weeks; PT group assessed at a mean GA of 41.3 weeks.	Neonates	25.4 (24–31)	755 (109)	Lund, Sweden 1984–1986	Hospital	Oxygen or medications needed during the time of testing; BW > 900 g.
Strand-Brodd et al,[54] 2011	**Visual following** [PD]<br>Gain of smooth pursuit (Gain), indicating the proportion of the object's movement that is followed by smooth pursuit; eye movement measured with EOG; stimulus: small happy face sketch; four test trials (35 sec each) indicating 4 conditions of object's movement (sinusoidal/triangular movement modulation × small/large amplitude)	3[L]<br>(T1: 2;<br>T2: 4)	28.5 (22–32)	1198	Uppsala, Sweden 2004–2007	Lab	Down syndrome; Bartter's syndrome; GA at birth > 32 weeks.
Sun & Buys,[55] 2012	**Focused attention**<br>"Sustained attention": persistence of effort, infant leaned in towards and fixated on an object while manipulating it (based on Ruff et al,[15] 1990; 5 trials, each with a different toy; 1 min each).	8	28.0<br>(1.9; < 32)	1008<br>(246)	Brisbane, Australia 1998–1999	Hospital	Health problems.
Wilcox et al,[56] 1996	**Habituation**<br>Time to reach criterion/trial length (fixed fixation time); criterion: accumulated 30 sec of looking at the stimulus or looking away from the display three times (2 sec each) after accumulated 10 sec of looking (odd-numbered trials).	4.56[L]<br>(T1: 2.54;<br>T2: 4.79;<br>T3: 6.56)	34.1<br>(0.8; 32–35)	2150<br>(240)	Texas, USA	Lab	Twins; abnormal BW for GA; known neurological insult; genetic and chromosomal abnormalities, infection, or disease.
Zuccarini et al,[57] 2017	**Focused attention**<br>MOE: exploration of the object using the hands or mouth while having continuous contact with it (based on Ruff et al,[8] 1984); 5 min session of free play with age-appropriate toys, with the mother's presence.	7.6[L]<br>(T1: 6.1;<br>T2: 9.1)	25.9<br>(1.5; 23–28)	803 (191)	Bologna, Italy	Hospital /Lab	GA at birth > 28; major cerebral damages detected by ultrasound and MRI (PVL; IVH > 2; hydrocephalus); congenital malformations; visual impairment (ROP > 2; blindness); hearing impairment.
Note. The measures obtained from each study are described. The age measure comprises both the full-term and the preterm groups. GA, BW and exclusion criteria refer to the preterm group, as the							

© 2021 Burstein et al. *JAMA Network Open*.

eTable 1. Characteristics of the studies included in the meta-analyses

Study	Included attention measure/s	Age in months	GA_{PT} (weeks) *M* (*SD*; Range)	BW_{PT} (g) *M* (*SD*)	Cohort	Setting	Exclusion criteria

full-term groups were sampled from typically developing and healthy infants.
BPD = bronchopulmonary dysplasia; BW = birthweight; GA = gestational age; IVH = intraventricular hemorrhage; PND = postnatal day; PT = preterm; PVL = periventricular leukomalacia; RDS = respiratory distress syndrome; ROP = retinopathy of prematurity; RT = response time; T = Time.
[L] Longitudinal study: measurements from all time points were pooled together to maintain an accurate sample size for each study.[58,59]
[Cor] Manuscript's authors have supplied additional data via personal correspondence.
[Med.1] Median and interquartile range were reported and were transformed into means and standard deviations according to equations 14 and 15 described by Wan et al.[60]
[Med.2] Median, minimum and maximum were reported and were transformed into mean and standard deviation according to equations 3 and 7 described by Wan et al.[60]
[PD] Plot digitizer[61] was employed to extract data from the manuscript.
[#] Both studies include the same sample, but report on different outcome measures.

eTable 2. Preterm Birth and the Development of Visual Attention during the First Two Years of Life: A Systematic Review and Meta-analysis (because of the size of the table we are hosting it here: https://data.mendeley.com/datasets/y6y85b5pty/2)

© 2021 Burstein et al. *JAMA Network Open*.

eAppendix 1. Modified Newcastle-Ottawa Scale for assessment of studies quality and risk of bias

The following coding guidelines were followed:

Selection
1) Representativeness of the preterm cohort
+ Truly or somewhat representative of the average characteristics of preterm-born infants in the community.
- Selected group of preterm-born infants (*e.g.*, specific hospital/clinic); no description of the derivation of the cohort.
2) Selection of the full-term cohort
+ Drawn from the same community as the preterm cohort.
- Drawn from a different source; no description of the derivation of the full-term cohort.
3) Ascertainment of prematurity
+ Secure record (*e.g.*, hospital record).
- Self report; no description.

Comparability
1) Comparable age
+ Study controls for corrected age.
- No description of group ages / a statistic test for comparing group ages / age correction for prematurity.
2) Other comparable factors
+ Study controls for gender, socioeconomic status, ethnicity or parental education.
- Significant differences between the cohorts in these factors.

Outcome
1) Independent assessment
+ Independent coders with an excellent inter-rater reliability statistic; computerized assessment.
- No description; non-excellent inter-rater reliability.
2) Blind assessment
+ Experimenters/coders were blind to the infant's group (preterm/full-term) or to the test condition.
- No description; experimenters/coders aware of the infant's group.
3) Data loss
+ Complete data from all subjects accounted for; subjects' data loss unlikely to introduce a bias – less than 20% of the data, or if an adequate reason for data loss is provided.
- More than 20% of the data lost and no description of those lost; no statement.

© 2021 Burstein et al. *JAMA Network Open*.

Study	Selection			Comparability		Outcome			Overall
	Representative	Control sample	Ascertainment of prematurity	Corrected age controlled	Other factors controlled	Assessment reliability	Blind assessment	Data loss	
Bonin et al,[1] 1998	−	+	+	−	+	+	−	+	5
Butcher et al,[2] 2002	−	−	+	−	−	+	−	+	3
Cherkes-Julkowski,[3] 1998	−	−	−	+	−	+	−	−	2
de Jong et al,[5] 2015	−	+	+	+	−	+	+	+	6
De Schuymer et al,[6] 2012	−	−	+	+	+	+	−	+	5
DiPietro et al,[7] 1992	−	−	+	+	−	+	+	+	5
Ferrari et al,[9] 1983	−	+	+	−	+	−	−	+	4
Foreman et al,[10] 1991	−	−	−	−	−	−	−	−	0
Forslund & Bjerre,[11] 1983	+	−	+	+	+	−	−	+	5
Friedman et al,[12] 1981	−	+	+	+	+	+	−	+	6
Harel et al,[13] 2011	−	−	−	+	+	−	+	+	4
Hodel et al,[14] 2017	−	+	+	+	+	+	+	+	7
Hunnius et al,[16] 2008	−	−	+	+	−	+	+	+	5
Kooiker et al,[17] 2019	−	−	+	+	+	+	−	+	6
Landry & Chapieski,[18] 1988	−	−	+	−	−	+	−	+	3
Landry et al,[19] 1985	−	−	+	+	+	−	−	+	4
Landry et al,[20] 1996	−	−	+	−	+	−	−	+	3
Lawson et al,[21] 1984	−	−	+	+	−	+	+	−	4
Leijon,[22] 1982	−	+	+	+	−	−	−	+	4
Lejeune et al,[23] 2019	−	+	+	+	+	+	−	+	6

© 2021 Burstein et al. *JAMA Network Open*.

Study	Selection			Comparability		Outcome			
	Representative	Control sample	Ascertainment of prematurity	Corrected age controlled	Other factors controlled	Assessment reliability	Blind assessment	Data loss	Overall
Lobo et al,[24] 2015	−	−	+	+	−	+	+	+	5
Loi et al,[25] 2017	−	−	+	+	+	+	+	+	6
Mash et al,[26] 1998	−	−	−	−	−	+	+	+	3
Ortiz-Mantilla et al,[27] 2008	−	−	+	+	+	+	+	+	6
Palmer et al,[28] 1982	−	+	+	+	−	−	−	+	4
Paludetto et al,[29] 1982	−	+	+	+	−	+	−	+	5
Pel et al,[30] 2016	−	−	+	+	−	+	+	+	5
Petkovic et al,[31] 2016	−	−	+	−	−	−	−	+	2
Petrie Thomas et al,[32] 2012	−	+	+	+	−	+	+	−	5
Pridham et al,[35] 2000	−	−	+	−	+	+	+	+	5
Provasi et al,[36] 2017	−	−	−	−	+	+	−	−	2
Reuner et al,[37] 2015	−	−	+	+	+	+	−	+	5
Ricci et al,[38] 2008	+	+	+	+	−	+	−	+	6
Riese,[39] 1987	−	−	+	−	+	+	−	+	4
Rose et al,[40] 1978	−	+	+	+	+	+	−	+	6
Rose et al,[41] 1979	−	−	−	+	+	+	+	+	5
Rose,[42] 1980	−	−	−	+	+	+	−	+	4
Rose,[43] 1983	−	−	−	+	+	+	+	+	5
Rose et al,[44] 1988	−	+	+	−	−	+	+	+	5
Rose et al,[45] 2001	−	+	+	+	+	+	−	+	6

© 2021 Burstein et al. *JAMA Network Open*.

Study	Selection			Comparability		Outcome			Overall
	Representative	Control sample	Ascertainment of prematurity	Corrected age controlled	Other factors controlled	Assessment reliability	Blind assessment	Data loss	
Rose et al,[46] 2002	−	+	+	+	+	+	−	+	**6**
Ross et al,[47] 1992	−	+	+	−	+	+	−	+	**5**
Ross-Sheehy et al,[48] 2017	−	−	+	+	−	+	−	+	**4**
Ruff et al,[49] 1982	−	+	+	−	−	+	+	+	**5**
Ruff et al,[8] 1984	−	+	+	+	−	+	−	+	**5**
Ruff,[50] 1986	−	−	−	+	−	−	−	+	**2**
Soares et al,[51] 2012	−	−	+	+	−	+	+	+	**5**
Spungen et al,[52] 1985	−	+	+	+	+	+	−	+	**6**
Stjernqvist & Svenningsen,[53] 1990	+	+	+	+	−	−	−	+	**5**
Strand-Brodd et al,[54] 2011	+	+	+	−	−	+	−	+	**5**
Sun & Buys,[55] 2012	−	+	+	+	+	−	−	+	**5**
Wilcox et al,[56] 1996	−	−	+	+	+	+	+	+	**6**
Zuccarini et al,[57] 2017	−	−	+	+	+	+	+	+	**6**

© 2021 Burstein et al. *JAMA Network Open*.

eAppendix 2. Description of the moderation analyses

Here we present a description of all of the factors that were considered in the moderation analyses. All of the significant moderators were conveyed in the main text.

Factors weighed in all analyses:
Mean age at test time (months); % of female infants; mean GA at birth of the preterm group; GA age group classification of the preterm group[62] (*i.e.*, extreme, very or moderate/late preterm birth); birthweight of the preterm group; publication year; location (USA vs. other); setting (*i.e.*, lab, hospital or home); cohort's birth era (before the antenatal corticosteroids and surfactant era [*i.e.*, year < 1990; Era I], after the introduction of antenatal corticosteroids and surfactants [*i.e.*, 1990<>2000; Era II], or after the introduction of further advancement in neonatal thermal, emotional and physical care strategies [*i.e.*, year > 2000; Era III]); NOS score (either as a continuous variable or a categorical one, with scores larger than 5 classified as high quality studies[63]); % of intraventricular hemorrhage in the preterm group.

Additional factors weighed in the *visual following* meta-analysis:
Age group (either neonates [k = 7] or infants [k = 4]); gaze tracking technology (either manual/videotaped examination by an experimenter or computerized eye tracking system [k = 2]); method of assessment in neonates (either the Brazelton Neonatal Behavioral Assessment Scale [BNBAS; k = 5] or other standardized tests [k = 2].

Additional factors weighed in the *latency to fixate* meta-analysis:
Gaze tracking technology (either manual/videotaped [k = 7] or computerized eye tracking system [k = 3]); type of measure (either response time [k = 5] or response time + % of correct fixations [k = 5]).

Additional factors weighed in the *habituation* meta-analysis:
The type of outcome measure (either time [k = 10] or trials [k = 3] to reach the habituation criterion); criterion type (either accumulating a predetermined fixation time [k = 7] or personally-customized, as a given frequency of decrement in looking time from baseline [k = 6]).

Additional factors weighed in the *novelty preference* meta-analysis:
The type of outcome measure (either the ratio [k = 11] of looking time at the novel stimulus to the total looking time [*i.e.*, novel + familiar] or the raw difference in looking time between the two stimuli [k = 6]); the method of stimuli presentation (either simultaneous presentation of the novel and familiar stimuli [k = 14] or presentation of the novel stimulus in a subsequent trial [k = 3]).

© 2021 Burstein et al. *JAMA Network Open*.

eAppendix 3. Moderation analysis in the latency to fixate meta-analysis

A moderation analysis was conducted to assess whether differences in latency to fixate between the preterm and term groups were associated with the cohort's birth era.

The analysis indicated that the cohort's birth era classification was associated with the differences in latency to fixate between the preterm-term groups ($Q_M = 6.38$, $df = 2$, $p = 0.041$). This suggests that when compared to studies with infants born prior to the corticosteroids and surfactant era ($\beta = -0.442$, $z = -2.34$, $p = 0.019$), in studies conducted with cohorts that were born between 1990 and 2000 better outcomes were more likely to be observed ($\beta = 0.598$, $z = 2.41$, $p = 0.016$). Regarding cohorts from Era III, neither increased risk nor advantage were observed when compared to cohorts born at Era I ($\beta = 0.231$, $z = 1.10$, $p = 0.27$); however, increased risk for deficits was found compared to cohorts from Era II ($\beta = -0.367$, $z = -1.98$, $p = 0.048$). A possible explanation for this finding is the inclusion of two studies[17,30] that involved infants born extremely preterm in Era III, while no such preterm GA group was examined in studies from previous eras. Indeed, upon removal of these studies from the analysis no increased risk for deficits was found in preterm infants born in Era III, when compared to Era II ($\beta = -0.282$, $z = -1.42$, $p = 0.15$).

A further inspection of studies involving cohorts from Era III, suggests that the differences between the preterm-term groups are still statistically significant in this era ($d = -0.211$; 95% CI, -0.39 to -0.03, $z = -2.28$, $p = 0.023$; $k = 6$), reflecting increased risk for deficits in latency to fixate ability following preterm birth. However, there are two factors that might explain this finding. First, as articulated above, Era III was the only Era including cohorts of infants born extremely preterm– a descriptive examination of the studies in Era III suggests the possibility of larger effect sizes in studies involving such populations (*i.e.*, $d = -0.53$,[30] -0.32[17]) when compared to studies involving very-to-late preterm born populations (*i.e.*, $d = 0.12$,[16] -0.01,[14], -0.18,[48] -0.42[6]). Upon removal of these two studies from the analysis no significant differences were observed between the preterm-term groups ($d = -0.126$; 95% CI, -0.35 to 0.10, $z = -1.11$, $p = 0.27$; $k = 4$). A second factor that could explain the findings is advancements in gaze assessment technologies, involving the implementation of computerized gaze tracking systems that were not utilized in previous eras (half of the studies in Era III utilized such equipment). A descriptive examination of the effect sizes in studies from Era III suggests the possibility of larger effect sizes in studies utilizing computerized gaze tracking systems (*i.e.*, $d = -0.42$,[6] -0.53,[30] and -0.32[17]) rather than manual/videotaped assessments (*i.e.*, $d = 0.12$,[16] -0.01,[14] and -0.18[48]). Taken together, it could be conjectured that the differences between the preterm-term groups in Era III are at least partially related to the inclusion of extremely preterm cohorts and the implementation of more sensitive methods for gaze assessment. The possibility of assessing the interaction between these two factors and birth era classification was not viable, as no studies from Eras I and II included them.

© 2021 Burstein et al. *JAMA Network Open*.

eAppendix 4. Moderation analysis in the habituation meta-analysis

A moderation analysis was conducted to assess whether differences in habituation between the preterm and term groups were associated with the cohort's birth era.

The analysis indicated that when considering all three birth era groups, only a trend towards significance was found for the differences in effect sizes (Q_M = 4.98, *df* = 2, *p* = 0.083). However, when considering the difference between studies conducted before 2000 to all other studies a significant moderation effect was found (Q_M = 4.77, *df* = 1, *p* = 0.029; *k* = 13; see eFigure 4), suggesting increased risk for deficits in habituation in preterm infants born before 2000 (β = -0.186, *z* = -2.3, *p* = 0.016; *d* = -0.186, 95% CI, -0.34 to -0.03, *p* = 0.016; *k* = 10), and lesser likelihood for such deficits in infants born in Era III (β = 0.318, *z* = 2.18, *p* = 0.029; *d* = 0.132, 95% CI, -0.11 to 0.37, *p* = 0.29; *k* = 3). Further, this association remained significant even when the only study[27] involving a group of infants born extremely preterm was removed from the analysis (Q_M = 3.91, *df* = 1, *p* = 0.048; *k* = 12).

eFigure 4. Graphical depiction of the moderation of the differences between the preterm and full-term groups in habituation by birth era

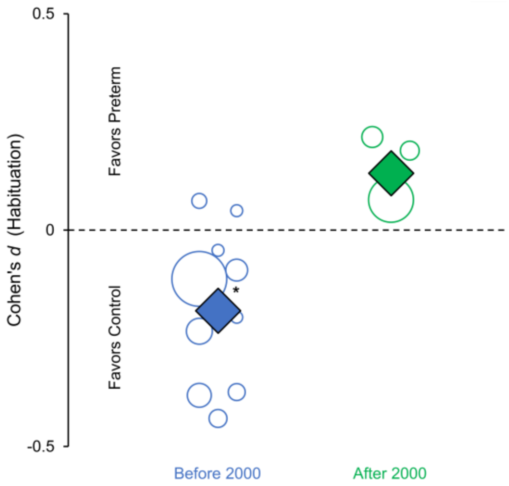

eFigure 4. Each circle represents an effect size of a distinct study, conducted either in cohorts born before (*k* = 10; depicted in blue) or after (*k* = 3; depicted in green) the year 2000. Diamonds represent the standardized total mean differences between the term-preterm groups at each birth era. The asterisk represents significant difference between the groups in studies involving cohorts born before the year 2000; no significant difference was observed in studies from the current era. Circle sizes are proportional to studies' weight. * $p < 0.05$

© 2021 Burstein et al. *JAMA Network Open*.

eAppendix 5. Additional analyses in the focused attention meta-analysis

Screening for outliers in the focused attention meta-analysis

A strong evidence of heterogeneity in effect sizes between studies was found in the focused attention meta-analysis (Q_{17} = 36.46, p = 0.004; I^2 = 53.4%). Additional screening has pinpointed two potential outliers with studentized residuals of -2.53[50] and -3.45.[55] A possible explanation for the deviant effect size in the study conducted by Holly Ruff[50] (a pioneer in the research of focused attention and exploratory behavior in infants) could be related to a partial report of the outcome measures. In the study by Sun & Buys,[55] a possible explanation for the deviant effect size is that the study included a cohort of infants born extremely preterm (see the subsequent moderation analysis for a further evaluation). Removal of the two potential outliers, slightly attenuated the pooled effect size, but did not annulled it (d = -0.17; 95% CI, -0.28 to -0.05; z = -2.88, p = 0.004). However, these studies were responsible for the heterogeneity, as no heterogeneity was evident following their removal (Q_{15} = 10.4, p = 0.79; I^2 = 0.0%).

Moderation analysis assessing whether birth era and GA group are associated with focused attention outcomes

In the focused attention meta-analysis, sufficient data were available to assess the interaction between cohort's birth era classification (*i.e.*, birth before or after 2000; as no studies with cohorts born before 1990 included infants born extremely preterm, only a comparison between these two eras was applicable) and GA group[62] (*i.e.*, extreme preterm [GA < 28 weeks], very preterm [GA of 28 to 32 weeks] or moderate/late preterm [GA of 32 to 37 weeks]). Classifications of the GA groups were based on the reported means and ranges for GA at birth.

The analysis indicated a significant interaction between cohort's birth era and GA group (Q_M = 11.6, df = 5, p = 0.04). To test whether birth after the year 2000 was associated with better focused attention performance in infants born extremely preterm, a *post hoc* analysis using a Dunnett's test (using the Bonferroni correction) with the before 2000-extreme preterm as the control group was conducted. The analysis indicated that infants born extremely preterm after 2000, had a lesser risk to show deficits in focused attention when compared to infants born extremely preterm before 2000 (β = 1.73, z = 2.87, p = 0.021). Further, in cohorts born before 2000, both the very- (β = 1.99, z = 3.52, p = 0.002) and moderate-late-preterm (β = 1.82, z = 3.11, p = 0.009) cohorts also had a lesser risk for deficits when compared to the extreme-preterm cohort that was born before 2000 (see eFigure 5 for a graphical depiction of the interaction).

Taken together the moderation analysis suggests that in cohorts born in Era III following extreme preterm birth, the risk for deficits in focused attention has attenuated– plausibly due to further advancements in neonatal care.

eFigure 5. Graphical depiction of the moderation of the differences between the preterm and full-term groups in focused attention by birth era and GA group

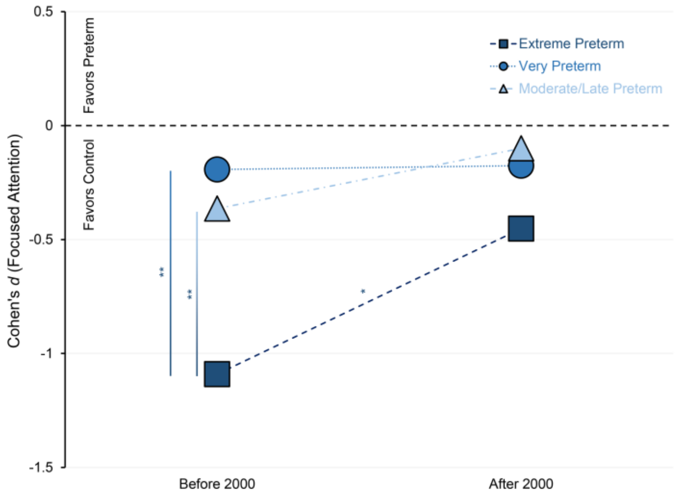

eFigure 5. Asterisks represent significant differences in effect sizes. ** $p < 0.01$ * $p < 0.05$

© 2021 Burstein et al. *JAMA Network Open*.

eReferences

1. Bonin M, Pomerleau A, Malcuit G. A longitudinal study of visual attention and psychomotor development in preterm and full-term infants during the first six months of life. *Infant Behav Dev*. 1998;21(1):103-118. doi:10.1016/S0163-6383(98)90057-1

2. Butcher PR, Kalverboer AF, Geuze RH, Stremmelaar EF. A longitudinal study of the development of shifts of gaze to a peripheral stimulus in preterm infants with transient periventricular echogenicity. *J Exp Child Psychol*. 2002. doi:10.1002/chem.201601060

3. Cherkes-Julkowski M. Learning disability, attention-deficit disorder, and language impairment as outcomes of prematurity: A longitudinal descriptive study. *J Learn Disabil*. 1998;31(3):294-306. doi:10.1177/002221949803100309

4. Krakow JB, Kopp CB. The effects of developmental delay on sustained attention in young children. *Child Dev*. 1983. doi:10.1111/j.1467-8624.1983.tb00535.x

5. de Jong M, Verhoeven M, van Baar AL. Attention capacities of preterm and term born toddlers: A multi-method approach. *Early Hum Dev*. 2015. doi:10.1016/j.earlhumdev.2015.08.015

6. De Schuymer L, De Groote I, Desoete A, Roeyers H. Gaze aversion during social interaction in preterm infants: A function of attention skills? *Infant Behav Dev*. 2012;35(1):129-139. doi:10.1016/j.infbeh.2011.08.002

7. DiPietro JA, Porges SW, Uhly B. Reactivity and Developmental Competence in Preterm and Full-Term Infants. *Dev Psychol*. 1992. doi:10.1037/0012-1649.28.5.831

8. Ruff HA, McCarton C, Kurtzberg D, Vaughan HG. Preterm Infants' Manipulative Exploration of Objects. *Child Dev*. 1984. doi:10.2307/1129985

9. Ferrari F, Grosoli M V., Fontana G, Cavazzuti GB. NEUROBEHAVIOURAL COMPARISON OF LOW-RISK PRETERM AND FULLTERM INFANTS AT TERM CONCEPTIONAL AGE. *Dev Med Child Neurol*. 1983. doi:10.1111/j.1469-8749.1983.tb13789.x

10. Foreman N, Fielder A, Price D, Bowler V. Tonic and phasic orientation in full-term and preterm infants. *J Exp Child Psychol*. 1991. doi:10.1016/0022-0965(91)90085-7

11. Forslund M, Bjerre I. Neurological assessment of preterm infants at term conceptional age in comparison with normal full-term infants. *Early Hum Dev*. 1983. doi:10.1016/0378-3782(83)90002-6

12. Friedman SL, Jacobs BS, Werthmann MWJ. Sensory processing in pre- and full-term infants in the neonatal period. In: Friedman SL, Sigman M, eds. *Preterm Birth and Psychological Development*. New York: Academic Press; 1981:159-178.

13. Harel H, Gordon I, Geva R, Feldman R. Gaze Behaviors of Preterm and Full-Term Infants in Nonsocial and Social Contexts of Increasing Dynamics: Visual Recognition, Attention Regulation, and Gaze Synchrony. *Infancy*. 2011. doi:10.1111/j.1532-7078.2010.00037.x

14. Hodel AS, Senich KL, Jokinen C, Sasson O, Morris AR, Thomas KM. Early executive function differences in infants born moderate-to-late preterm. *Early Hum Dev*. 2017;113:23-30. doi:10.1016/j.earlhumdev.2017.07.007

15. Ruff HA, Lawson KR, Parrinello R, Weissberg R. Long-Term Stability of Individual Differences in Sustained Attention in the Early Years. *Child Dev*. 1990. doi:10.1111/j.1467-8624.1990.tb02760.x

16. Hunnius S, Geuze RH, Zweens MJ, Bos AF. Effects of preterm experience on the developing visual system: A longitudinal study of shifts of attention and gaze in early infancy. *Dev Neuropsychol*. 2008. doi:10.1080/87565640802101508

17. Kooiker MJG, Swarte RMC, Smit LS, Reiss IKM. Perinatal risk factors for visuospatial attention and processing dysfunctions at 1year of age in children born between 26 and 32weeks. *Early Hum Dev*. 2019;130:71-79. doi:10.1016/j.earlhumdev.2019.01.015

18. Landry SH, Chapieski ML. Visual attention during toy exploration in preterm infants: Effects of medical risk and maternal interactions. *Infant Behav Dev*. 1988;11(2):187-204. doi:10.1016/S0163-

© 2021 Burstein et al. *JAMA Network Open*.

6383(88)80005-5

19. Landry SH, Leslie NA, Fletcher JM, Francis DJ. Visual attention skills of premature infants with and without intraventricular hemorrhage. *Infant Behav Dev*. 1985. doi:10.1016/0163-6383(85)90020-7

20. Landry SH, Garner PW, Swank PR, Baldwin CD. Effects of maternal scaffolding during joint toy play with preterm and full-term infants. *Merrill Palmer Q*. 1996;42(2):177-199.

21. Lawson KR, Ruff HA, McCarton-Daum C, Kurtzberg D, Vaughan Jr. HG. Auditory–visual responsiveness in full-term and preterm infants. *Dev Psychol*. 1984;20(1):120-127. doi:10.1037/0012-1649.20.1.120

22. Leijon I. Assessment of behaviour on the Brazelton scale in healthy preterm infants from 32 conceptional weeks until full-term age. *Early Hum Dev*. 1982. doi:10.1016/0378-3782(82)90130-X

23. Lejeune F, Lordier L, Pittet MP, et al. Effects of an early postnatal music intervention on cognitive and emotional development in preterm children at 12 and 24 months: Preliminary findings. *Front Psychol*. 2019. doi:10.3389/fpsyg.2019.00494

24. Lobo MA, Kokkoni E, Cunha AB, Galloway JC. Infants Born Preterm Demonstrate Impaired Object Exploration Behaviors Throughout Infancy and Toddlerhood. *Phys Ther*. 2015. doi:10.2522/ptj.20130584

25. Loi EC, Vaca KEC, Ashland MD, Marchman VA, Fernald A, Feldman HM. Quality of caregiver-child play interactions with toddlers born preterm and full term: Antecedents and language outcome. *Early Hum Dev*. 2017. doi:10.1016/j.earlhumdev.2017.10.001

26. Mash C, Quinn PC, Dobson V, Narter DB. Global influences on the development of spatial and object perceptual categorization abilities: Evidence from preterm infants. *Dev Sci*. 1998. doi:10.1111/1467-7687.00017

27. Ortiz-Mantilla S, Choudhury N, Leevers H, Benasich AA. Understanding language and cognitive deficits in very low birth weight children. *Dev Psychobiol*. 2008;50(2):107-126. doi:10.1002/dev.20278

28. Palmer PG, Dubowitz LMS, Verghote M, Dubowitz V. Neurological and neurobehavioural differences between preterm infants at term and full-term newborn infants. *Neuropediatrics*. 1982. doi:10.1055/s-2008-1059620

29. Paludetto R, Mansi G, Rinaldi P, et al. Behaviour of preterm newborns reaching term without any serious disorder. *Early Hum Dev*. 1982. doi:10.1016/0378-3782(82)90073-1

30. Pel JJM, Dudink J, Vonk M, Plaisier A, Reiss IKM, van der Steen J. Early identification of cerebral visual impairments in infants born extremely preterm. *Dev Med Child Neurol*. 2016. doi:10.1111/dmcn.13115

31. Petkovic M, Chokron S, Fagard J. Visuo-manual coordination in preterm infants without neurological impairments. *Res Dev Disabil*. 2016. doi:10.1016/j.ridd.2016.01.010

32. Petrie Thomas JH, Whitfield MF, Oberlander TF, Synnes AR, Grunau RE. Focused attention, heart rate deceleration, and cognitive development in preterm and full-term infants. *Dev Psychobiol*. 2012;54(4):383-400. doi:10.1002/dev.20597

33. Ruff HA, Lawson KR. Development of Sustained, Focused Attention in Young Children During Free Play. *Dev Psychol*. 1990. doi:10.1037/0012-1649.26.1.85

34. Lawson KR, Ruff HA. Focused attention: Assessing a fundamental cognitive process in infancy. In: *Biobehavioral Assessment of the Infant.* New York, NY, US: The Guilford Press; 2001:293-311.

35. Pridham K, Becker P, Brown R. Effects of infant and caregiving conditions on an infant's focused exploration of toys. *J Adv Nurs*. 2000. doi:10.1046/j.1365-2648.2000.01448.x

36. Provasi J, Lemoine-Lardennois C, Orriols E, Morange-Majoux F. Do Preterm Infants Perceive Temporal Synchrony? An Analysis with the Eye-Tracking System. *Timing Time Percept*. 2017. doi:10.1163/22134468-00002089

37. Reuner G, Weinschenk A, Pauen S, Pietz J. Cognitive development in 7- to 24-month-old

extremely/very-to-moderately/late preterm and full-term born infants: The mediating role of focused attention. *Child Neuropsychol*. 2015;21(3):314-330. doi:10.1080/09297049.2014.899571

38. Ricci D, Cesarini L, Romeo DMM, et al. Visual function at 35 and 40 weeks' postmenstrual age in low-risk preterm infants. *Pediatrics*. 2008;122(6):e1193-e1198. doi:10.1542/peds.2008-1888

39. Riese ML. Longitudinal assessment of temperament from birth to 2 years: A comparison of full-term and preterm infants. *Infant Behav Dev*. 1987. doi:10.1016/0163-6383(87)90022-1

40. Rose SA, Gottfried AW, Bridger WH. Cross-modal transfer in infants: Relationship to prematurity and socioeconomic background. *Dev Psychol*. 1978. doi:10.1037/0012-1649.14.6.643

41. Rose SA, Gottfried AW, Bridger WH. Effects of haptic cues on visual recognition memory in fullterm and preterm infants. *Infant Behav Dev*. 1979. doi:10.1016/S0163-6383(79)80008-9

42. Rose SA. Enhancing visual recognition memory in preterm infants. *Dev Psychol*. 1980. doi:10.1037/0012-1649.16.2.85

43. Rose SA. Differential rates of visual information processing in full-term and preterm infants. *Child Dev*. 1983. doi:10.1111/j.1467-8624.1983.tb00539.x

44. Rose SA, Feldman JF, Wallace IF. Individual differences in infants' information processing: reliability, stability, and prediction. *Child Dev*. 1988. doi:10.1111/j.1467-8624.1988.tb01488.x

45. Rose SA, Feldman JF, Jankowski JJ. Attention and recognition memory in the 1st year of life: a longitudinal study of preterm and full-term infants. *Dev Psychol*. 2001. doi:10.1037/0012-1649.37.1.135

46. Rose SA, Feldman JF, Jankowski JJ, Caro DM. A longitudinal study of visual expectation and reaction time in the first year of life. *Child Dev*. 2002;73(1):47-61. doi:10.1111/1467-8624.00391

47. Ross G, Tesman J, Auld PA, Nass R. Effects of subependymal and mild intraventricular lesions on visual attention and memory in premature infants. *Dev Psychol*. 1992;28(6):1067-1074. doi:10.1037/0012-1649.28.6.1067

48. Ross-Sheehy S, Perone S, Macek KL, Eschman B. Visual orienting and attention deficits in 5- and 10-month-old preterm infants. *Infant Behav Dev*. 2017. doi:10.1016/j.infbeh.2016.12.004

49. Ruff HA, Lawson KR, Kurtzberg D, Mccarton-daum C, Vaughan HG. Visual following of moving objects by full-term and preterm infants. *J Pediatr Psychol*. 1982. doi:10.1093/jpepsy/7.4.375

50. Ruff HA. Attention and organization of behavior in high-risk infants. *J Dev Behav Pediatr*. 1986;7(5):298-301. doi:10.1097/00004703-198610000-00004

51. Soares D de A, von Hofsten C, Tudella E. Development of exploratory behavior in late preterm infants. *Infant Behav Dev*. 2012. doi:10.1016/j.infbeh.2012.09.002

52. Spungen LB, Kurtzberg D, Vaughan HG. Patterns of looking behavior in full-term and low birth weight infants at 40 weeks post-conceptional age. *J Dev Behav Pediatr*. 1985. doi:10.1097/00004703-198510000-00011

53. Stjernqvist K, Svenningsen NW. Neurobehavioural Development At Term Of Extremely Low-Birthweight Infants. *Dev Med Child Neurol*. 1990;32(8):679-688. doi:10.1111/j.1469-8749.1990.tb08428.x

54. Strand-Brodd K, Ewald U, Grönqvist H, et al. Development of smooth pursuit eye movements in very preterm infants: 1. General aspects. *Acta Paediatr*. 2011;100(7):983-991. doi:10.1111/j.1651-2227.2011.02218.x

55. Sun J, Buys N. A comparison of sustained attention in very preterm and term infants. *Int J Child Adolesc health*. 2012;5(3):291-300.

56. Wilcox T, Nadel L, Rosser R. Location memory in healthy preterm and full-term infants. *Infant Behav Dev*. 1996;19(3):309-323. doi:10.1016/S0163-6383(96)90031-4

57. Zuccarini M, Guarini A, Savini S, et al. Object exploration in extremely preterm infants between 6 and 9 months and relation to cognitive and language development at 24 months. *Res Dev Disabil*. 2017.

© 2021 Burstein et al. *JAMA Network Open*.

doi:10.1016/j.ridd.2017.06.002

58. Borenstein M, Hedges L V, Higgins JPT, Rothstein HR. *Introduction to Meta-Analysis*. John Wiley & Sons; 2011.

59. Card NA. *Applied Meta-Analysis for Social Science Research*. New York: The Guilford Press; 2012.

60. Wan X, Wang W, Liu J, Tong T. Estimating the sample mean and standard deviation from the sample size, median, range and/or interquartile range. *BMC Med Res Methodol*. 2014. doi:10.1186/1471-2288-14-135

61. Huwaldt JA, Steinhorst S. Plot digitizer. *URL http//plotdigitizer sourceforge net*. 2013.

62. WHO. *Born Too Soon: The Global Action Report on Preterm Birth*.; 2012. doi:http://whqlibdoc.who.int/publications/2012/9789241503433_eng.pdf

63. Twilhaar ES, Wade RM, De Kieviet JF, Van Goudoever JB, Van Elburg RM, Oosterlaan J. Cognitive outcomes of children born extremely or very preterm since the 1990s and associated risk factors: A meta-analysis and meta-regression. *JAMA Pediatr*. 2018. doi:10.1001/jamapediatrics.2017.5323

© 2021 Burstein et al. *JAMA Network Open*.
